# Immune intrinsic escape signature stratifies prognosis, characterizes the tumor immune microenvironment, and identifies tumorigenic PPP1R8 in glioblastoma multiforme patients

**DOI:** 10.3389/fimmu.2025.1577920

**Published:** 2025-08-06

**Authors:** Ran Du, Lijun Jing, Denggang Fu

**Affiliations:** ^1^ Department of Neurology Intensive Care Unit, The First Affiliated Hospital of Zhengzhou University, Zhengzhou, China; ^2^ College of Medicine, Medical University of South Carolina, Charleston, SC, United States

**Keywords:** glioblastoma multiforme, immune escape related genes, outcome, immune microenvironment, PPP1R8

## Abstract

**Background:**

Glioblastoma (GBM) is a highly aggressive brain tumor with poor prognosis and limited response to immunotherapy. Immune escape-related genes (IERGs) are increasingly recognized as critical regulators of tumor progression and immune evasion. However, their prognostic value in GBM remains unclear. This study aims to evaluate the clinical relevance of IERGs and develop a predictive gene signature to guide prognosis and characterize the tumor immune microenvironment (TIME).

**Methods:**

We performed a comprehensive analysis of IERGs using the TCGA GBM dataset. Prognostic IERGs were identified through univariate Cox regression, and a multivariate Cox model was used to develop a prognostic signature. Risk scores (IEScore) were calculated to classify patients into high- and low-risk groups. The signature was validated in two independent GBM cohorts. Its prognostic independence was assessed after adjusting for clinicopathological features. Receiver operating characteristic (ROC) analysis confirmed the signature’s reliability. TIME analysis was carried out using multiple deconvolution algorithms. Additionally, functional assays including CCK8, cell cycle, and apoptosis assays were conducted on PPP1R8-silenced U251 cells using CRISPR/Cas9 technology

**Results:**

Thirty-six IERGs were associated with GBM outcomes, with 20 linked to poor survival and 16 to better outcomes. Key genes, including STAT2, IFNGR2, and PPP1R8, formed a robust prognostic signature. High-risk patients had significantly poorer overall survival (OS) compared to low-risk patients. The signature showed strong predictive power with AUC values of 0.68, 0.73, and 0.76 for 2-, 3-, and 5-year survival, respectively. Validation in two independent cohorts confirmed its robustness. Immune cell infiltration analysis revealed distinct patterns in high- and low-risk groups, with the high-risk group showing a more aggressive and immunosuppressive tumor microenvironment. The signature also effectively stratified low-grade glioma patients across four independent datasets. Knockout of PPP1R8 in GBM cells using CRISPR/Cas9 inhibited cell proliferation and increased apoptosis.

**Conclusion:**

The IERGs-based signature offers reliable prognostication for GBM, validated across multiple datasets. It can guide patient stratification and inform therapeutic decisions for GBM and potentially low-grade gliomas (LGG). Furthermore, we identify PPP1R8 as a key regulator of GBM cell proliferation and growth, providing insights into the immune microenvironment’s role in GBM progression.

## 1 Introduction

Glioblastoma multiforme (GBM) is recognized as one of the most aggressive and treatment-resistant brain tumors, with a median survival of approximately 15 months despite maximal surgical resection, radiotherapy, and chemotherapy (1, 2). This dire prognosis highlights an urgent need for innovative therapeutic strategies and biomarkers that can enhance patient outcomes. Central to the challenge of effective treatment is the tumor’s remarkable ability to evade immune surveillance, a phenomenon that has gained significant attention in cancer research.

Immune evasion refers to the mechanisms by which tumor cells avoid detection and destruction by the host immune system (3, 4). Dunn et al. first articulated the concept of immunoediting, which includes the selection of less immunogenic tumor variants and the alteration of the tumor microenvironment to favor immune tolerance (5). Tumor cells utilize multiple mechanisms to evade immune responses, including the downregulation of major histocompatibility complex (MHC) molecules, the secretion of immunosuppressive cytokines, and the expression of immune checkpoint proteins (6, 7). For example, the interaction between programmed cell death protein 1 (PD-1) on T cells and its ligand PD-L1 on tumor cells plays a critical role in inhibiting T cell activation and promoting tumor growth (8). In GBM, increased PD-L1 expression has been associated with poorer patient outcomes, underscoring its role in immune evasion (9–12). The tumor immune microenvironment significantly influences immune responses and facilitates immune evasion in GBM (13, 14). TME is characterized by a complex interplay of immune cells, stromal cells, and extracellular matrix components, contributing to an immunosuppressive milieu. Tumor-associated macrophages (TAMs), regulatory T cells (Tregs), and myeloid-derived suppressor cells (MDSCs) are prevalent in GBM and secrete cytokines and chemokines that inhibit effective anti-tumor immunity (15–17). For instance, TAMs produce TGF-β and IL-10, which further suppress T cell activity and promote tumor growth (18). The presence of these immunosuppressive cells has been correlated with reduced patient survival and treatment resistance (19, 20).

Research efforts have increasingly focused on elucidating specific immune evasion-related gene signatures associated with prognosis in GBM and other malignancies. We and various studies have employed genomic and transcriptomic approaches to identify immune-related genes that correlate with patient outcomes (21–23). A robust immune-related gene signature that significantly associated with overall survival (OS) in GBM patients was proposed, highlighting the potential of these genes as prognostic biomarkers (24). Similarly, we and other groups have established several signatures that highlight the significance of immune-related gene sets in predicting treatment responses and clinical outcomes across different cancers (25–28). Additional studies emphasized the importance of characterizing the immune landscape in tumors to identify relevant immune signatures for prognostication (29–31).

In recent years, the development of immune therapies, including immune checkpoint inhibitors and CAR T-cell therapies, has opened new avenues for treating GBM (32). Clinical trials have demonstrated that checkpoint inhibitors, such as nivolumab and pembrolizumab, can restore anti-tumor immune responses, though their effectiveness in GBM has been variable (33, 34). Additionally, CAR T-cell therapies targeting specific antigens expressed on GBM cells have shown promise in early-phase trials, offering hope for more effective treatments (35, 36). However, the complex immunosuppressive TME poses significant challenges to the efficacy of these therapies, underscoring the need for better predictive models and combinatory approaches.

Despite these advancements, there remains a critical need to establish comprehensive immune evasion signatures that can more accurately predict prognosis and treatment responses in GBM. Current signatures often focus on a limited set of genes and may not fully capture the complexity of the immune landscape within tumors. By developing a more robust immune evasion-related gene signature, researchers can better elucidate the mechanisms driving immune suppression in GBM and identify novel therapeutic targets. Such signatures could facilitate the stratification of patients for immunotherapy, ensuring that those most likely to benefit receive appropriate treatment (37, 38). Moreover, integrating genomic data with insights into the tumor microenvironment will enhance our understanding of the dynamic interactions between tumor cells and immune components (39, 40). This approach may reveal new biomarkers and therapeutic avenues that could improve patient outcomes. As immunotherapy continues to evolve, one of the persistent challenges in GBM treatment is the tumor’s capacity to evade immune surveillance. In this context, our study aimed to construct and validate a robust immune evasion-related gene signature (IEScore) through integrative bioinformatic analyses of transcriptomic data from The Cancer Genome Atlas (TCGA) and other public datasets. Unlike prior studies that focused on limited gene panels or single datasets, we employed a multi-layered strategy that combined multivariate survival modeling across multiple cohorts, tumor immune microenvironment (TIME) characterization, single-cell transcriptomic resolution, and functional *in vitro* validation. Specifically, we (1) established a prognostic gene signature associated with immune escape in GBM, (2) validated its prognostic significance across independent GBM and LGG cohorts, (3) linked the signature to immune cell infiltration patterns using deconvolution algorithms, (4) resolved the cellular expression of signature genes using single-cell RNA sequencing, and (5) experimentally confirmed the tumor-promoting role of PPP1R8 using CRISPR/Cas9 knockout in GBM cells. These efforts collectively provide a translational framework that not only advances our understanding of GBM immune evasion but also supports clinical risk stratification and therapeutic target development. A schematic overview of the study design is presented in **Supplementary Figure S1**.

## 2 Methods and materials

### 2.1 Data acquisition and pre-processing

Gene expression data and clinical information for glioma patients were obtained from TCGA via the GlioVis GBM platform (http://gliovis.bioinfo.cnio.es/) (21). RNA-seq data were processed using normalized read counts and log2 transformation. Additional datasets, including those from the Gene Expression Omnibus (GEO), were also obtained from the GlioVis and utilized for analysis. Clinical and RNA-seq data from GEO and TCGA were collected, addressing missing values and standardizing data for consistency.

For the training dataset, 525 TCGA samples were used (41), while external validation cohorts included the CGGA (n=237) (42) and LeeY (n=191) (43) GBM datasets. The demographic and clinical characteristics of these cohorts are summarized in **Supplementary Table S1**. This study adhered to the guidelines of the Declaration of Helsinki (2013 update).

### 2.2 Identification of overall survival - related immune escape-related genes

Immune escape-related genes were extracted from the TCGA-GBM dataset (**Supplementary Table S2**) (44). Clinical features were integrated with gene expression data to form a unified dataset. Univariate Cox regression analysis was performed to identify IERGs significantly associated with OS in GBM, using a threshold of p < 0.05.

### 2.3 Pathway enrichment analysis

Gene Ontology (GO) and Kyoto Encyclopedia of Genes and Genomes (KEGG) pathway analyses were conducted using the clusterProfiler R package (45). These analyses identified key biological processes and pathways associated with OS-related IERGs in GBM.

### 2.4 Model construction and validation

We developed a prognostic model based on IERGs using survival data and gene expression profiles from the TCGA GBM cohort. The analysis was conducted in R using the “survival” package.

First, univariate Cox proportional hazards regression was performed to identify IERGs significantly associated with OS. To prevent overfitting and improve model interpretability, we applied the least absolute shrinkage and selection operator (LASSO) regression to select the most relevant prognostic genes and construct the final multigene signature.

For each patient, a risk score was calculated as a linear combination of the expression levels of the selected genes, weighted by their corresponding LASSO coefficients. We then determined the median risk score across all patients in the training cohort (TCGA), and used this median value as a cutoff to divide patients into high-risk and low-risk groups:

High-risk group: Patients with risk scores above the median, typically associated with shorter survival. Low-risk group: Patients with risk scores below the median, generally showing longer survival.

To evaluate the prognostic significance of the model, we used Kaplan-Meier survival analysis and compared the OS between the two risk groups using the log-rank test.

To assess the model’s predictive accuracy, we conducted receiver operating characteristic (ROC) curve analysis using the “pROC” R package. The area under the curve (AUC) was calculated to quantify the model’s ability to discriminate between high- and low-risk patients.

For external validation, we applied the same risk score formula and median cutoff to two independent GBM datasets: CGGA (n=237) and LeeY (n=191). The performance of the model in these cohorts was assessed using the same methods described above to confirm its robustness and generalizability.

### 2.5 Time-dependent predictive performance comparison with established models

We evaluated the prognostic accuracy of our proposed IEScore in comparison to two published interferon-related signatures in GBM: IFNG_sig (46) and IFNGrGS (47). Cox proportional hazards models were fitted for each signature using the same training dataset, with OS and event status as outcomes.

Time-dependent AUCs were calculated at 0.5-year intervals from 0.5 to 5 years using the Score() function from the riskRegression R package. For each model, AUCs and corresponding 95% confidence intervals were estimated via nonparametric bootstrapping (B = 1000), allowing assessment of discriminative performance over time.

To compare predictive accuracy across models, pairwise comparisons of time-dependent AUCs were conducted. Differences in AUCs (ΔAUC), along with 95% confidence intervals and p-values, were calculated using 1,000 bootstrap iterations to evaluate statistical significance at each time point.

### 2.6 Predictive utility of the signature for low-grade glioma

To evaluate the prognostic potential of the IERGs signature in LGG, survival data were obtained from TCGA-LGG (n=509) (48), Gravendeel_set (n=116) (49), Kamoun_set (n=136) (50), and Rembrandt_set (n=161) (51). Patients were stratified into high- and low-risk groups based on the median risk score. Survival differences between the two groups were assessed using the log-rank test, and the predictive performance was further evaluated using ROC curve analysis as described above.

### 2.7 Analysis of low-grade and high-grade gliomas

To further explore the relationship between IERGs and glioma malignancy, we extended our analysis to include both LGG and GBM samples from TCGA databases. Gene expression and corresponding clinical data, including OS, were obtained from the TCGA-LGG and TCGA-GBM datasets. We applied the established IERG-based prognostic model to the combined LGG and GBM dataset. Risk scores were calculated for each patient based on the expression of the signature genes and their associated model coefficients. Patients were stratified into high- and low-risk groups using the median risk score as the cutoff. Kaplan–Meier survival curves were generated to compare OS between the two groups, and statistical significance was assessed using the log-rank test.

To examine the distribution of glioma grades within each risk group, we compared the frequencies of LGG and GBM across the high-risk and low-risk populations. The association between tumor grade and risk group classification was evaluated using the Chi-squared test.

### 2.8 Analysis of immune infiltration and clinical correlations

Immune cell infiltration within the tumor immune microenvironment was assessed using multiple computational deconvolution algorithms across high- and low-risk patient groups. The xCell algorithm (52) was employed to generate a comprehensive profile of immune and stromal cell populations. To achieve a more detailed quantification of specific immune cell subsets, CIBERSORT (53) analysis was performed. Additionally, immune infiltration estimations were further validated using TIMER (54) and EPIC (55) deconvolution methods, providing complementary perspectives on the immune landscape in both risk groups.

### 2.9 Signature gene expression in the TME using single-cell RNA sequencing

To further investigate the expression of signature genes in the TME of GBM patients, we obtained the processed single-cell RNA sequencing dataset (GSE131928) (56). Cell populations were identified using the Seurat R package, following previously established methods (57). The expression patterns of the signature genes (STAT2, IFNGR2, PPP1R8) were analyzed in different cell populations and visualized using Uniform Manifold Approximation and Projection (UMAP).

### 2.10 Cell culture and reagents

U251 cells were cultured in EMEM medium supplemented with 2mM glutamine, 1% non-essential amino acids, 1mM aodium pyruvate, 10% fetal bovine serum, at 37°C in a humidified atmosphere containing 5% CO2.

### 2.11 CRISPR/Cas9-mediated knockout of PPP1R8 in U251 cells

PPP1R8 knockout in U251 cells was achieved using CRISPR/Cas9 technology. The CRISPR-Cas9/gRNA ribonucleoprotein (CRISPR-RNP) complex was delivered via electroporation using the Neon NxT Electroporation System (Thermo Fisher). Electroporation parameters were set to a pulse voltage of 1400 V, a pulse width of 20 ms, and three pulses at a cell density of 1 x 10^6^ cells/mL. The sgRNA sequence targeting exon 3 of the human PPP1R8 gene was GGACCCGAGAGCAAGACUGG AUGGUAAAGUCACACAAAUC, with a specific cleavage site spanning positions 27,838,750 to 27,838,776.

Knockout efficiency was validated using RT-qPCR. Briefly, total mRNA was extracted from U251 cells using Trizol reagent. Reverse transcription was performed using the HiScript II Q RT SuperMix Kit (Vazyme, Cat: R223-01), and RT-qPCR was conducted with the Taq Pro Universal SYBR qPCR Master Mix kit (Vazyme, Cat: Q712-02). The primer pairs: PPP1R8, forward: CTTCAGCGGAGGACTCTACG; reverse:GGGGCAAGGTTTGGGTATGG, and reference control GAPDH primer pairs were previously described (27). Gene expression was quantified using 2^–ΔΔCt^ method.

In addition, western blot was used to assess PPP1R8 knockout efficiency. Cells were lysed in RIPA buffer (Thermo Fisher, cat: 89900) supplemented with protease and phosphatase inhibitors (CST, cat: #5872). Equal amounts of protein (20-30 µg) were separated by SDS-PAGE and transferred to PVDF membranes (Millipore, cat:IPVH00010). Membranes were blocked with 5% non-fat milk in TBST and incubated overnight at 4°C with rabbit polyclonal anti-PPP1R8 antibody (1:1000; Abcam, A96784). After washing, membranes were probed with HRP-conjugated secondary antibody (1:5000; CST) for 1 hour at room temperature. Signals were detected using ECL substrate (Bio-Rad) and imaged with a ChemiDoc system (Bio-Rad).

### 2.12 Cell proliferation assay

The effect of PPP1R8 knockout on U251 cell proliferation was evaluated using the Cell Counting Kit-8 (CCK-8) assay. Forty-eight hours after gene knockout, U251 cells were seeded into 96-well plates at a density of 2 x 10^5^ cells per well and incubated for 24 hours at 37°C with 5% CO2 to allow cell attachment. Following incubation, 10 µL of CCK-8 reagent was added to each well, and the cells were further incubated for 4 hours under the same conditions. Absorbance was measured at 450 nm using a microplate reader to determine cell viability.

### 2.13 Cell cycle and apoptosis assays

For cell cycle analysis, U251 cells were cultured in EMEM medium supplemented with 2 mM glutamine, 1% non-essential amino acids, 1 mM sodium pyruvate, and 10% fetal bovine serum at 37°C in a humidified atmosphere containing 5% CO2 for 48 hours. After incubation, cells were harvested, fixed, and stained with Ki-67 and DAPI. Approximately 10,000 cells per sample were analyzed using flow cytometry to determine the distribution across different phases of the cell cycle, following the manufacturer’s protocol (FITC and 7-AAD kit).

For apoptosis analysis, wild-type and PPP1R8-knockdown U251 cells were seeded into 6-well plates at a density of 5 x 10^5^ cells per well and cultured for 24 hours. Cells were then dissociated with pancreatin, washed with 1 x PBS, and stained using an Annexin V-FITC/PI apoptosis detection kit (Cat#: C1062M, Beyotime Biotechnology, China) according to the manufacturer’s instructions. Flow cytometry (FongCyte, China) was used to assess apoptotic cell distribution.

### 2.14 Statistical analysis

Several R software packages were employed for statistical analysis and data visualization. The “survival” package was used for Cox regression analysis and Kaplan-Meier survival curve generation. The differences in survival between groups were assessed with the “survfit” function. The “pROC” package was used to create ROC curves and compute AUC values. A p-value < 0.05 was considered indicative of statistical significance.

## 3 Results

### 3.1 Prognostic relevance of immune escape related genes in adult GBM patients

**Figure 1:**
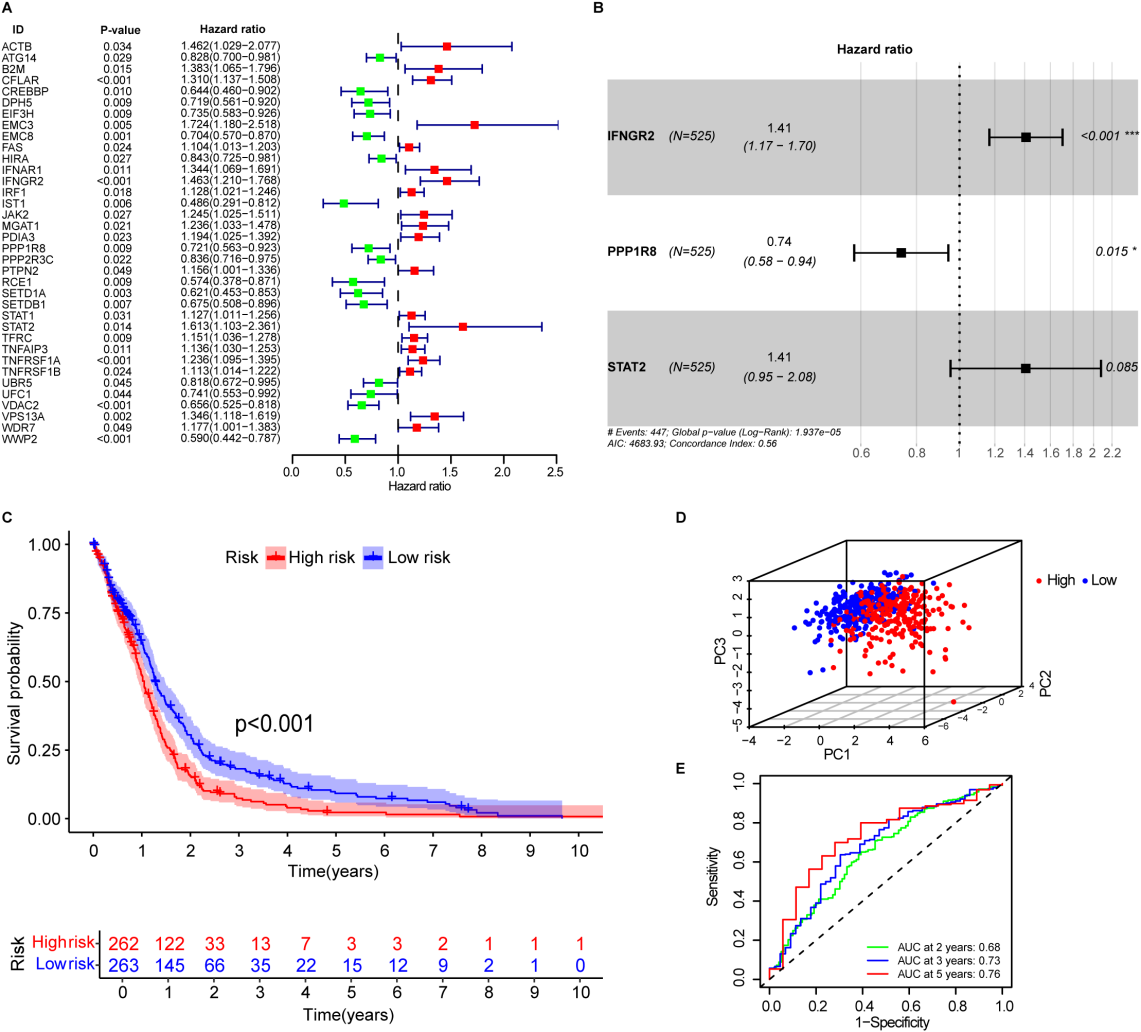
Development of immune escape related genes associated prognostic signature. **(A)** Forest plot showing immune escape-related genes associated with OS in GBM patients. **(B)** The signature consists of three key immune escape-related genes: IFNGR2, STAT2, and PPP1R8. **(C)** Kaplan–Meier survival curves for high- and low-risk patients in the training cohort, stratified by IEScore. **(D)** Principal component analysis (PCA) of patients in the low- and high-risk groups based on the IEScore. **(E)** Area under the curve (AUC) values of the signature for 2-, 3-, and 5-year OS prediction.

To evaluate the clinical relevance of immune escape-related genes (IERGs) in GBM patients, we performed univariate Cox regression analysis using the TCGA-GBM dataset. This analysis identified 36 IERGs significantly associated with patient prognosis (**Figure 1A**). Among them, elevated expression of 20 genes including ACTB, B2M, EMC3, IFNAR1, IFNGR2, and STAT2 were associated with poorer OS. In contrast, higher expression of the remaining 16 IERGs correlated with improved patient outcomes.

To further explore the biological functions of the prognostic IERGs, we conducted functional enrichment analysis, which revealed that these genes were primarily involved in interferon-mediated signaling pathways and cellular responses to type II interferon, interferon-beta, and extrinsic apoptotic signaling (**Supplementary Figure S2A**). Subsequent pathway analysis confirmed that the IL-6/JAK/STAT3 pathway, interferon response, apoptosis, and inflammatory response were among the most significantly enriched pathways (**Supplementary Figure S2B**, **Supplementary Table S3**).

### 3.2 Construction of the prognostic signature based on OS-related IERGs

The 36 prognostic IERGs were further analyzed using stepwise multivariate Cox proportional hazards regression to identify the minimal gene set that provides the strongest predictive power for patient outcomes. This analysis resulted in a three-gene prognostic signature comprising STAT2, IFNGR2, and PPP1R8 (**Figure 1B**). The risk score was calculated using the following formula:


Immune Escape related−Signature Score(IEScore)=[Expression level of STAT2*(0.3423)]+[Expression level of IFNGR2*(0.3436)]+[Expression level of PPP1R8*(−0.3015)]

Each patient’s IEScore was determined using the three-gene signature, and patients were divided into high- and low-risk groups based on the median IEScore. Kaplan-Meier survival analysis showed that high-risk patients had significantly shorter OS compared to low-risk patients (**Figure 1C**, p<0.001). Principal component analysis (PCA) demonstrated a clear separation between the two groups (**Figure 1D**). Expression analysis revealed that IFNGR2 and PPP1R8 were upregulated in GBM tissues relative to adjacent normal tissues, while STAT2 expression was comparable between the two (**Supplementary Figure S3A**). In the high-risk group, IFNGR2 and STAT2 were elevated, whereas PPP1R8 was more highly expressed in the low-risk group (**Supplementary Figure S3B**).

The predictive accuracy of the signature was supported by time-dependent ROC analysis, with AUC values of 0.68, 0.73, and 0.76 for 2-, 3-, and 5-year survival, respectively (**Figure 1E**), indicating good prognostic performance.

### 3.3 Validation of the prognostic signature in independent GBM datasets

**Figure 2:**
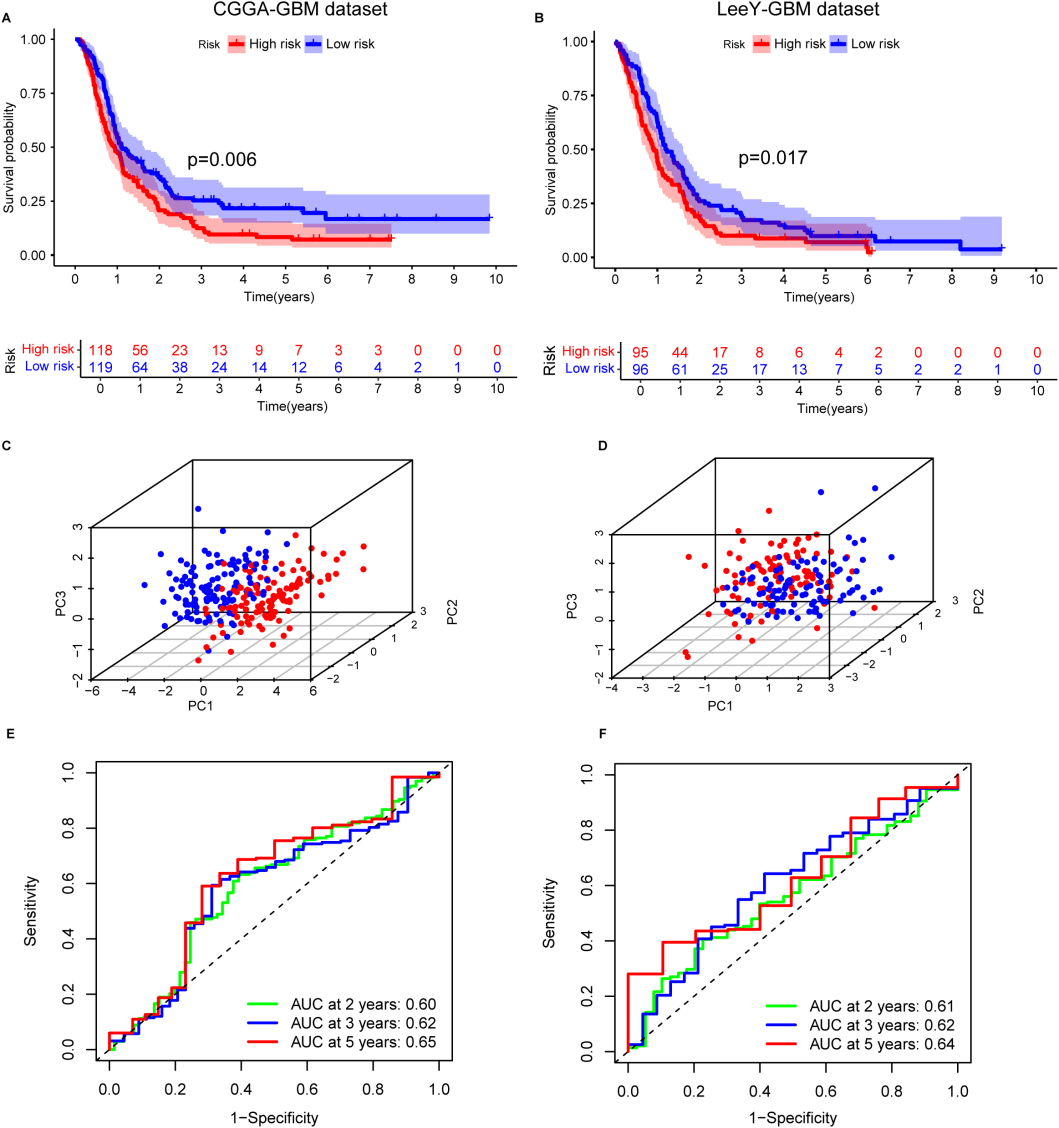
Validation of the immune escape-related gene (IERG) signature in two independent GBM cohorts. **(A)** Kaplan–Meier survival curves comparing OS between high- and low-risk groups in the CGGA-GBM cohort, stratified by IEScore. **(B)** Kaplan–Meier survival curves for high- and low-risk patients in the LeeY-GBM cohort. **(C)** Principal component analysis (PCA) illustrating the distribution of patients in high- and low-risk groups in the CGGA-GBM cohort. **(D)** PCA showing the separation of risk groups in the LeeY-GBM cohort. **(E)** Time-dependent ROC curves showing the predictive performance of the signature for 2-, 3-, and 5-year OS in the CGGA-GBM cohort. **(F)** ROC curves displaying the AUC values of the signature for 2-, 3-, and 5-year survival prediction in the LeeY-GBM cohort.

To assess the robustness and reproducibility of the prognostic signature in GBM, we applied the IEScore formula to two independent publicly available GBM cohorts-CGGA-GBM (n=237) and the LeeY dataset (n=191)—comprising a total of 428 GBM patients. Patients were stratified into low- and high-risk groups based on the median IEScore. In both cohorts, low-risk patients exhibited significantly longer OS than high-risk patients (**Figures 2A, B**).

In the CGGA cohort, the time-dependent ROC analysis demonstrated good predictive performance, with AUC values for 2-, 3-, and 5-year survival all exceeding 0.6 (**Figure 2C**). PCA confirmed clear separation between high- and low-risk groups (**Figure 2E**). Similar patterns of risk stratification, PCA clustering, and AUC values were observed in the LeeY dataset (**Figures 2D, F**), further supporting the model’s reliability.

Consistent with the training dataset, STAT2 and IFNGR2 expression levels were elevated in the high-risk group across both validation cohorts. In contrast, PPP1R8 expression was higher in the low-risk group, matching the expression pattern observed in the training set (**Supplementary Figures S3C, D**). These findings confirm that the three-gene prognostic signature enables effective and reproducible risk stratification of GBM patients across independent microarray-based datasets.

### 3.4 Time-dependent discriminatory ability of the models

To assess the prognostic discrimination of our proposed immune evasion score (IEScore) in comparison with two interferon-related signatures (IFNG_sig and IFNGrGS) in glioblastoma (GBM), we computed time-dependent area under the curve (AUC) values at 0.5-year intervals from 0.5 to 5 years post-diagnosis. As shown in **Supplementary Table S4_sheet A** and **Supplementary Figure S4**, the IEScore demonstrated a steadily increasing predictive performance over time, with the AUC rising from 53.3% (95% CI: 47.2–59.5%) at 0.5 years to 73.4% (62.1–84.8%) at 5 years. In contrast, the IFNG_sig maintained a moderate but relatively stable performance throughout the time course, with AUC values ranging from 52.3% to 59.5%. The IFNGrGS signature showed the lowest discriminatory ability, with AUCs declining to as low as 46.4% (34.4–58.4%) at 4 years and only reaching 54.1% (39.4–68.8%) at 5 years.

To further quantify the differences in predictive accuracy between models, we conducted pairwise time-dependent AUC comparisons using the riskRegression package (**Supplementary Table S4_sheet B**). The IEScore consistently outperformed the IFNGrGS signature across all time points from 1.5 to 5 years, with statistically significant differences (e.g., ΔAUC = −21.9% at 3.5 years, p < 0.001). Compared to the IFNG_sig, the IEScore also showed superior performance at multiple later time points, particularly between 2.5 and 3.5 years (ΔAUC = −9.9% and −14.8%, p = 0.037 and 0.010, respectively). However, the magnitude of this difference decreased slightly at the later time points.

Collectively, these findings suggest that the IEScore demonstrates sustained prognostic value in GBM patients, particularly beyond the 2-year mark, when compared to existing interferon-related signatures. Its consistent performance over time, as evidenced by both absolute AUC values and pairwise comparisons, highlights its potential as a clinically meaningful tool for long-term prognosis. These results also reflect the inherent heterogeneity of GBM across different patient cohorts, warranting further investigation to validate and strengthen the comparative observations.

### 3.5 Association of the prognostic signature with patients’ clinical features

**Figure 3:**
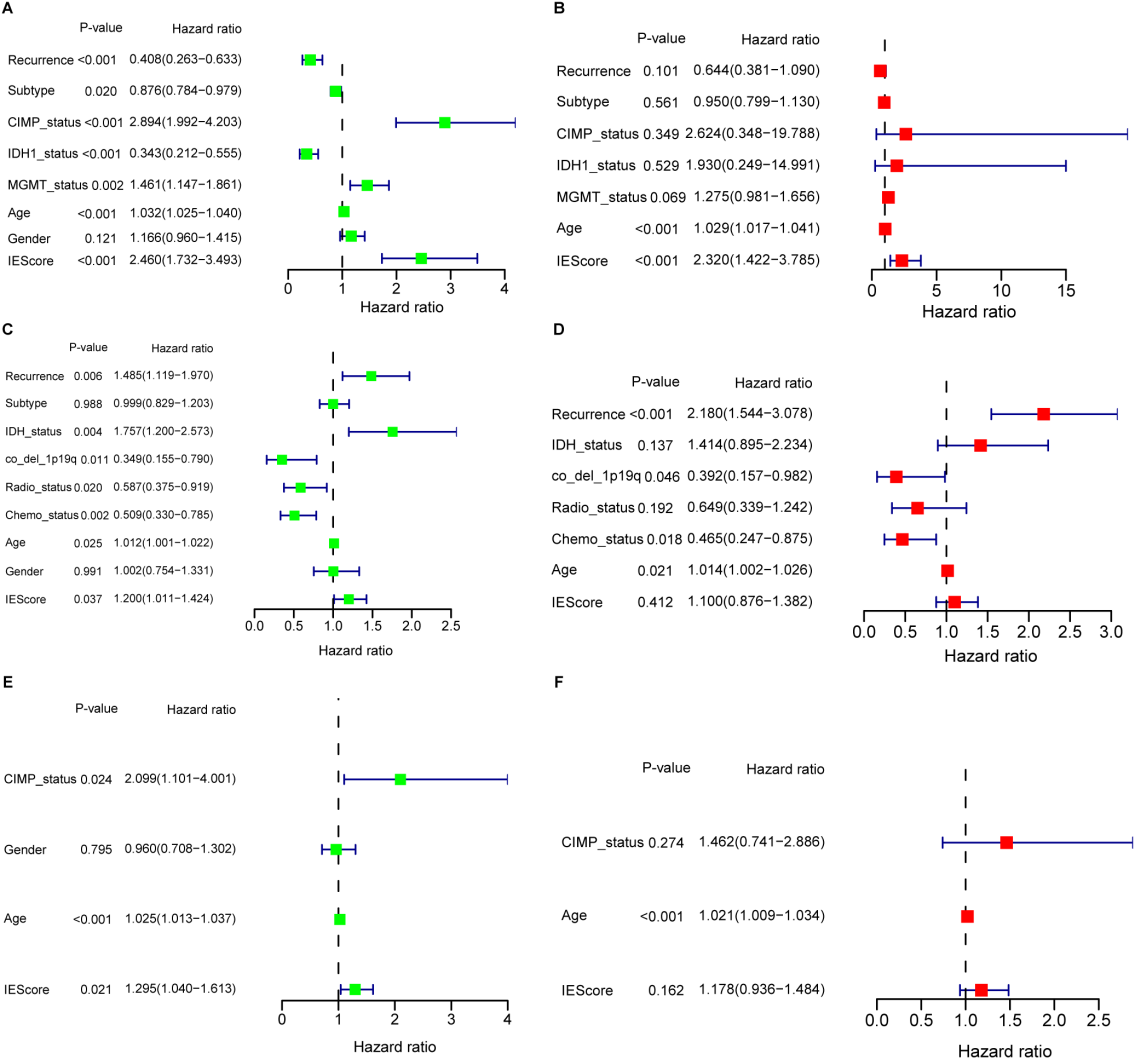
Univariate and multivariate Cox regression analyses evaluating the prognostic value of the IERG-based signature across GBM cohorts. **(A)** Univariate Cox regression analysis of the IERG signature and clinical features in the TCGA-GBM cohort. **(B)** Multivariate Cox regression analysis in the TCGA-GBM cohort, demonstrating the independent prognostic value of the signature. **(C)** Univariate Cox regression analysis in the CGGA-GBM cohort. **(D)** Multivariate Cox regression analysis in the CGGA-GBM cohort. **(E)** Univariate Cox regression analysis in the LeeY-GBM cohort. **(F)** Multivariate Cox regression analysis in the LeeY-GBM cohort.

Clinicopathological features such as O6-methylguanine (O6-MeG)-DNA methyltransferase (MGMT) promoter methylation status (58) and oncogenic mutations (59) are known to influence GBM progression and patient’s prognosis. To evaluate the prognostic independence of the IEScore, We conducted univariate Cox regression analysis using the TCGA GBM dataset. The results showed that high IEScore, CIMP status, MGMT status, and older age correlated with reduced OS, while patients with primary GBM subtype and IDH1 mutations exhibited favorable survival (**Figure 3A**). Multivariate Cox regression analysis further demonstrated that the IEScore remained a significant independent prognostic factor after adjusting for other clinical variables, including treatment (**Figure 3B**). Similar trends were observed in the CGGA and LeeY validation cohorts (**Figures 3C, E**). However, in multivariate analyses of these validation sets, IEScore did not reach statistical significance for OS prediction, likely due to the limited number of surviving patients, given the aggressive nature of GBM (**Figures 3D, F**). In addition, clinical and molecular features varied notably across the training (TCGA) and validation cohorts (CGGA and LeeY), as summarized in **Supplementary Table S1**. For instance, the CGGA cohort includes a higher proportion of recurrent GBM cases and more IDH1-mutant and G-CIMP-positive tumors compared to TCGA, while treatment information (radiotherapy and chemotherapy) is only available in CGGA. The LeeY cohort lacks recurrence and treatment data but differs in subtype composition. These inter-cohort differences may contribute to the reduced multivariate significance of IEScore observed in external validation datasets. Furthermore, older age was consistently associated with worse prognosis, aligning with previous studies that link aging with immunosuppression and reduced efficacy of immunotherapies in glioblastoma (60–62).

To continue to compare IEScores among patients with different clinical features, we found that IEScores did not show difference among patients with various disease status, age, and gender (**Supplementary Figures S5A–C**). Mesenchymal subtypes have the highest IEScore than classical and proneural subtypes (**Supplementary Figure S5D**). Patients with non-CIMP status have higher IEScore than those in CIMP status (**Supplementary Figure S5E**), which was in line with previous studies indicated that glioblastomas with CIMP-positive status often show widespread methylation across these CpG islands, which can lead to silencing of tumor suppressor genes and is associated with better prognosis (63). In addition, patients with wild IDH1 or non-MGMT have higher IEScore (**Supplementary Figures S5F, G**), which also confirmed previous reports (58, 59). This was also validated in CGGA GBM dataset (**Supplementary Figures S6A–G**), and in addition, patients without or receiving chemotherapy or radiotherapy showed no difference risk score (**Supplementary Figures S6H, I**). In LeeY validation set, IEScores distribution in partial clinical features were confirmed due to limited available clinical features (**Supplementary Figures S7A–D**).

### 3.6 Predictive potential of the signature for low-grade glioma

To extend the applicability of the prognostic signature to LGG, which are more frequently diagnosed in younger patients, we evaluated its performance in four independent LGG cohorts with available OS data: TCGA-LGG, Gravendeel, Kamoun, and Rembrandt datasets. These datasets represent diverse populations, providing a solid foundation to assess the robustness and generalizability of the prognostic signature.

**Figure 4:**
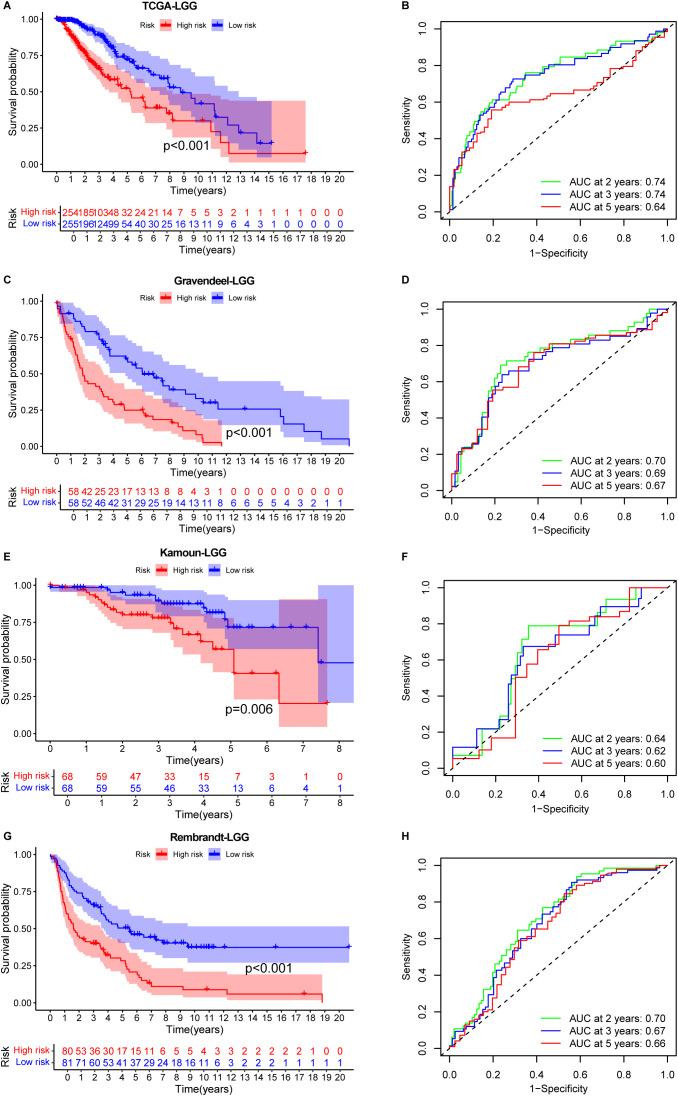
Prognostic performance of the IERG-based signature in low-grade glioma (LGG) cohorts. **(A)** Kaplan–Meier survival curves comparing OS between high- and low-risk patients in the TCGA-LGG cohort. **(B)** Time-dependent ROC curves showing 2-, 3-, and 5-year AUC values for the signature in the TCGA-LGG cohort. **(C)** Kaplan–Meier survival analysis in the Gravendeel-LGG cohort stratified by IEScore. **(D)** ROC curves showing the predictive accuracy of the signature at 2, 3, and 5 years in the Gravendeel-LGG cohort. **(E)** Kaplan–Meier survival curves for risk groups in the Kamoun-LGG cohort. **(F)** ROC analysis illustrating the prognostic performance of the signature in the Kamoun-LGG cohort. **(G)** Kaplan–Meier survival curves in the Rembrandt-LGG cohort comparing high- and low-risk patients. **(H)** Time-dependent AUC values for 2-, 3-, and 5-year OS prediction in the Rembrandt-LGG cohort.

When applied to the TCGA-LGG dataset, the signature effectively stratified patients into high- and low-risk groups. Patients in the low-risk group showed a significantly better OS compared to those in the high-risk group (**Figure 4A**). The signature’s predictive accuracy was further supported by an AUC value of 0.74 for 2- and 3-year survival (**Figure 4B**), indicating strong prognostic power.

Consistent findings were observed in the three additional validation cohorts. In the Gravendeel (**Figure 4C**), Kamoun (**Figure 4E**), and Rembrandt (**Figure 4G**) datasets, the signature effectively stratified patients by survival outcomes. The AUC values for 2-, 3-, and 5-year survival were all above 0.6 (**Figures 4D, F, H**), further supporting the reliability and reproducibility of the IERGs signature across multiple LGG cohorts.

Collectively, these results suggest that the IERGs signature holds strong potential as a prognostic tool for monitoring OS in LGG patients. Its integration into clinical practice could facilitate more accurate risk stratification, enabling personalized treatment strategies and improved decision-making in the management of LGG.

### 3.7 Association of the prognostic signature with the malignancies

To explore the potential association between the immune escape-related prognostic signature and glioma malignancy, we integrated transcriptomic data from both LGG and glioblastoma GBM patients in the TCGA cohort, following batch effect correction. IEScores were calculated for each patient using the established signature, and individuals were stratified into high- and low-risk groups based on the median IEScore. Kaplan–Meier survival analysis revealed that high-risk patients had significantly shorter OS compared to low-risk patients (**Supplementary Figure S8A**), confirming the prognostic value of the signature across glioma grades. However, the distribution of LGG and GBM cases between high- and low-risk groups did not show a significant difference (**Supplementary Figure S8B**), suggesting that the signature may not directly reflect tumor grade or malignancy. Furthermore, differential expression analysis of the IERGs between LGG and GBM revealed no significant differences (data not shown). This lack of distinction may indicate that the immune escape-related gene signature captures immune-related prognostic features that are independent of histological grade. It is also possible that certain immune evasion mechanisms are shared across glioma subtypes, contributing similarly to patient outcomes regardless of tumor grade.

### 3.8 Tumor immune microenvironment analysis

**Figure 5:**
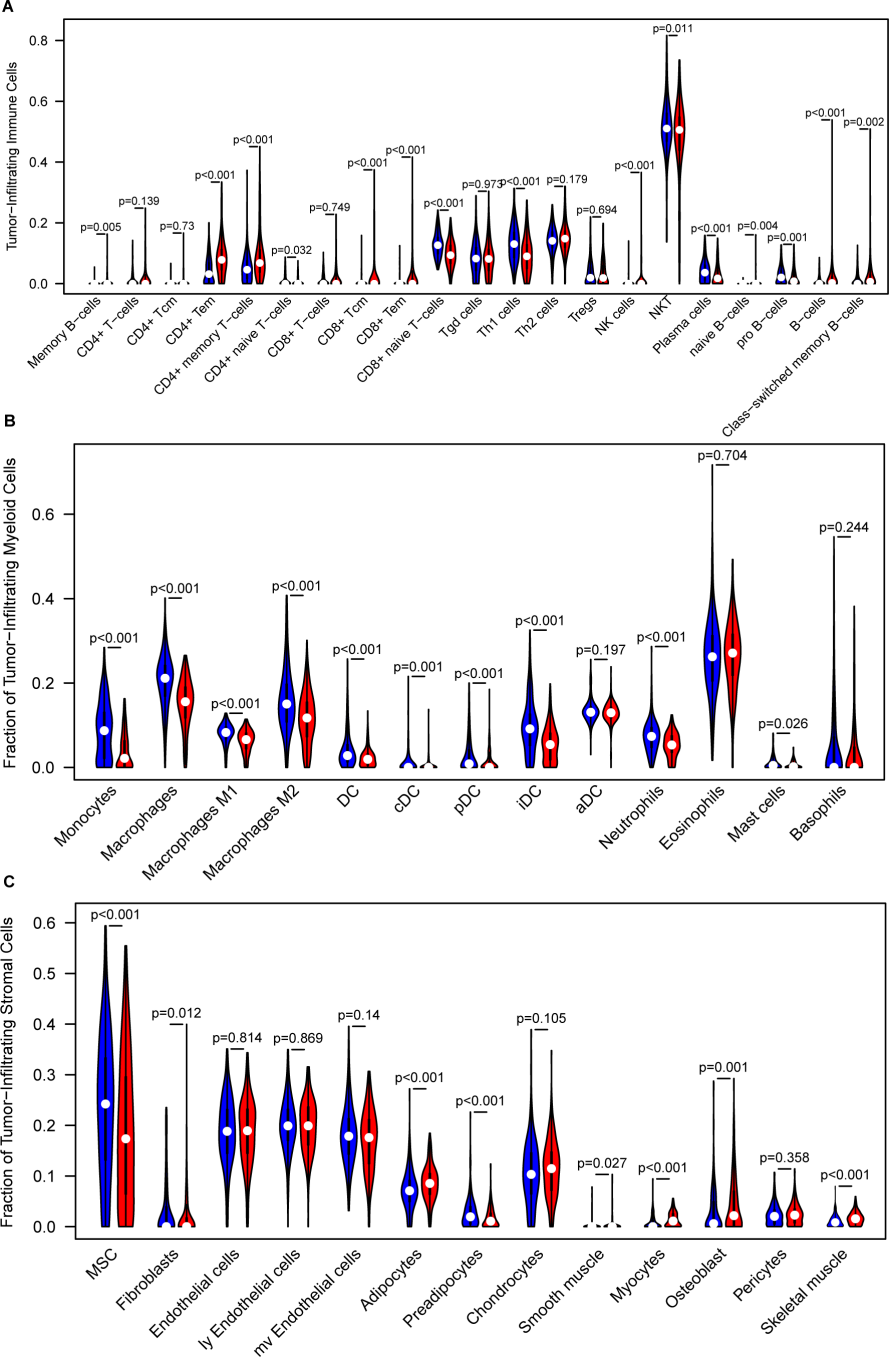
Analysis of the tumor immune microenvironment. **(A)** Comparison of infiltrated immune cell subsets between low- and high-risk groups, analyzed using xCell in the training GBM dataset. **(B)** Comparison of infiltrated myeloid cell subsets between low- and high-risk groups, analyzed using xCell in the training GBM dataset. **(C)** Comparison of infiltrated stromal cell subsets between low- and high-risk groups, analyzed using xCell in the training GBM dataset.

The recognition of the dual role of the tumor immune microenvironment (TIME) in anti-tumor immunity has led to significant advancements in tumor immunotherapy (64). To better understand the TIME landscape in different risk groups, we analyzed the immune cell infiltration profiles using multiple deconvolution algorithms, including xCell and CIBERSORT. The xCell analysis revealed that, in the low-risk group, immune cell subsets such as CD4^+^ effector memory T cells, CD8^+^ T cells, CD8^+^ effector memory T cells, NK cells, and B cells were notably elevated. In contrast, the high-risk group exhibited higher levels of the naive CD8^+^ T cells, NKT cells, and plasma cells (**Figure 5A**). For infiltrating myeloid cells, the high-risk group showed significantly higher levels of macrophages, M1/M2 macrophages, various dendritic cell (DC) subsets, neutrophils, and mast cells compared to the low-risk group (**Figure 5B**). These results suggest a distinct immune microenvironment that could be associated with the more aggressive tumor phenotype observed in the high-risk group. Additionally, when analyzing infiltrating stromal cells, we observed that the high-risk group had significantly higher proportions of mesenchymal stem cells and preadipocytes, along with lower frequencies of fibroblasts, adipocytes, smooth muscle cells, myocytes, osteoblasts, and skeletal muscle cells, compared to the low-risk group (**Figure 5C**). These findings were further validated in independent datasets, including the CGGA GBM validation set (**Supplementary Figures S9A–C**) and the LeeY GBM validation set (**Supplementary Figures S10A–C**).

Furthermore, CIBERSORT deconvolution analysis further supported these observations, confirming the differential immune cell infiltrates in the TCGA, CGGA, and LeeY GBM datasets (**Supplementary Figure S11**).

To further validate the immune landscape associated with the IEScore, we applied two additional immune deconvolution algorithms, EPIC and TIMER, to estimate immune cell proportions across TCGA_GBM, CGGA_GBM, and LeeY_GBM cohorts. In all three datasets, we observed consistent and significant differences in several immune subsets between high- and low-risk patients. Specifically, TIMER analysis revealed higher proportions of CD4^+^ T cells, and macrophages, neutrophils and myeloid dendritic cells in the high-IEScore group as compared to those patients in the low-IEScore group (**Supplementary Figure S12A**). EPIC analysis showed a similar trend, with high-IEScore patients enriched in CD4^+^ T cells, macrophages, cancer-associated fibroblasts and endothelial cells were more abundant in high-IEScore patients (**Supplementary Figure S12B**). These consistent trend across multiple algorithms and independent cohorts support the immune-suppressive nature of high-IEScore tumors and further corroborate the robustness of our immune stratification model.

**Figure 6:**
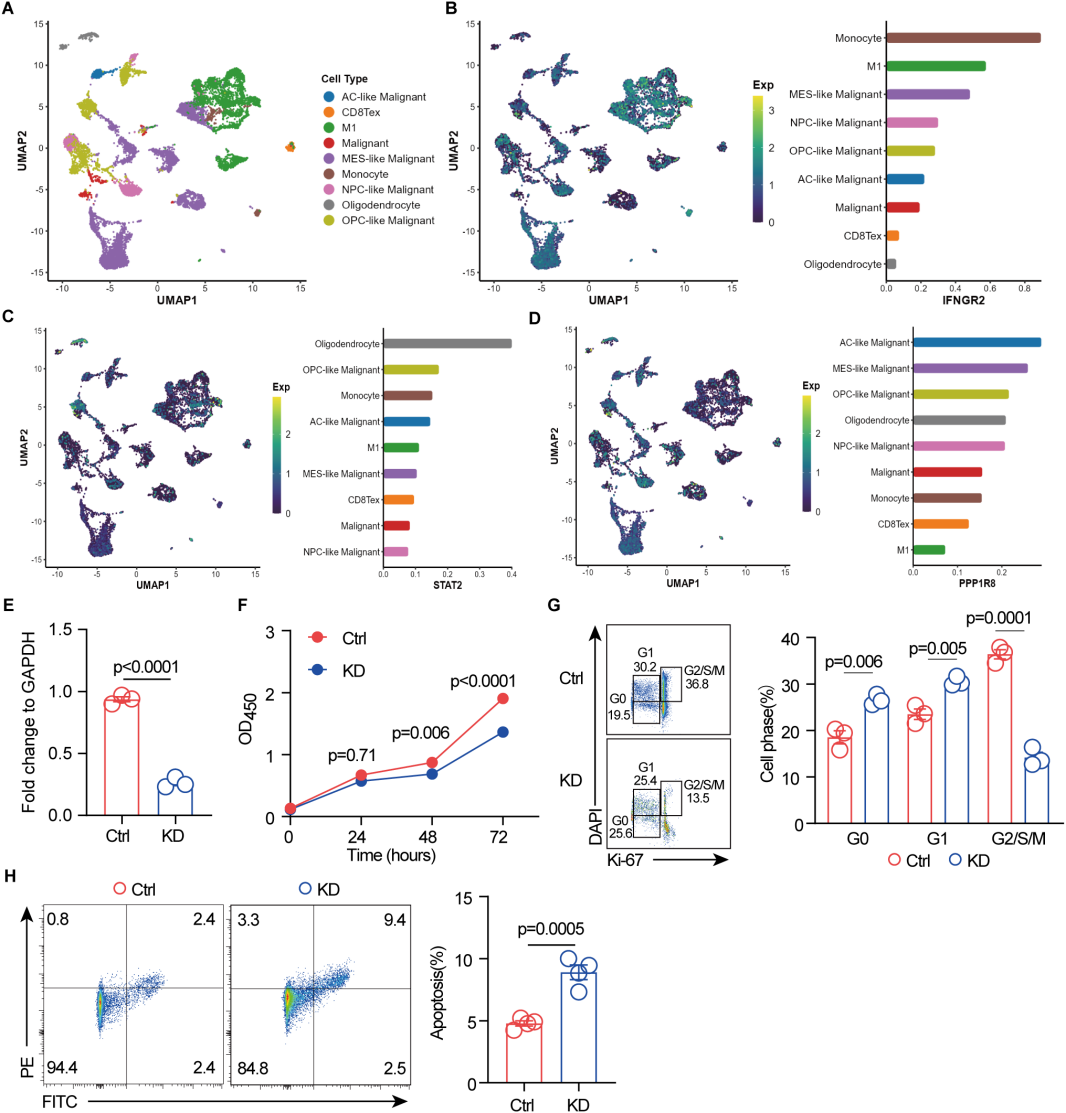
Single-cell RNA sequencing reveals signature gene expression in the GBM tumor microenvironment, and PPP1R8 deficiency suppresses proliferation while promoting apoptosis. **(A)** Identification of nine distinct cell populations using the Seurat R package. **(B)** UMAP visualization of IFNGR2 expression across the nine cell populations. **(C)** UMAP visualization of STAT2 expression across the nine cell populations. **(D)** UMAP visualization of PPP1R8 expression across the nine cell populations. **(E)** Validation of PPP1R8 knockdown in CRISPR/Cas9-edited U251 cells compared to control cells. **(F)** Cell proliferation assay (CCK8) comparing PPP1R8 knockdown and control U251 cells. **(G)** Cell cycle analysis of PPP1R8 knockdown and control U251 cells. **(H)** Apoptosis analysis of PPP1R8 knockdown and control U251 cells.

To further investigate the expression of these three signature genes in the tumor microenvironment at the single-cell level, we first identified nine major cell clusters using the Seurat R package: AC-like malignant cells, CD8-exhausted T cells, M1 macrophages, malignant cells, MES-like malignant cells, monocytes, NPC-like malignant cells, oligodendrocytes, and OPC-like malignant cells (**Figure 6A**). We then assessed the distribution of these genes across these cell populations and observed distinct expression patterns. IFNGR2 was predominantly expressed in monocytes, M1 macrophages, and MES-like malignant cells (**Figure 6B**), aligning with previous findings that IFNGR2 is preferentially expressed by monocyte/macrophage populations within the glioma microenvironment (65). STAT2 exhibited the highest expression in oligodendrocytes, followed by OPC-like malignant cells (**Figure 6C**). This observation is consistent with previous studies highlighting the involvement of the JAK-STAT pathway in oligodendrocyte maturation, a process crucial for the function of these myelinating cells in the central nervous system (66). The role of PPP1R8 in GBM remains unclear. Our analysis revealed its predominant expression in malignant cell populations, including AC-like, MES-like, OPC-like, and NPC-like malignant cells (**Figure 6D**). This finding suggests a potential oncogenic role for PPP1R8 in GBM, warranting further investigation.

### 3.9 PPP1R8 deficiency inhibits proliferation of GBM cells

The roles of key signature genes, including STAT2 and IFNGR2 (65, 67–71) in the tumorigenesis of GBM have been well established, with some emerging as potential therapeutic targets. STAT2 plays a critical role in the host STAT2/type I interferon axis, which controls tumor growth. Moreover, a novel risk signature of interferon response genes has been identified as a prognostic indicator and is correlated with immune infiltration in glioblastoma. Similarly, IFNGR2 has been implicated in glioma prognosis and responsiveness to immune checkpoint blockade. Studies have shown that upregulation of the canonical interferon-gamma signaling pathway is associated with glioblastoma progression, and its expression in the tumor microenvironment significantly impacts overall survival in pediatric diffuse midline glioma (DMG) patients (47, 70–73). While the role of PPP1R8 has been investigated in LGG (74), where its deficiency has been shown to decrease cell proliferation and the proportion of cells in the G2/M and S phases while increasing the G0/G1 population, its role in GBM, a highly aggressive glioma, remains unexplored. To assess PPP1R8 expression, we examined its mRNA levels across the NCI-60 cancer cell line panel, including GBM cell lines. Our analysis revealed that PPP1R8 expression was elevated in GBM cell lines, particularly U251 (**Supplementary Figure S13A**). To further explore its functional role, we utilized CRISPR/Cas9 technology to knock down PPP1R8 in U251 cells, and its deficiency was validated using RT-qPCR (**Figure 6E**) and western blot (**Supplementary Figure S13B**). The impact of PPP1R8 knockdown (KD) on cell proliferation was assessed using the CCK-8 assay, which demonstrated a significant reduction in cell number following PPP1R8 depletion (**Figure 6F**). Furthermore, cell cycle analysis revealed that PPP1R8 KD decreased the G2/S/M population and increased the proportion of cells in the G0/G1 phase, suggesting an impairment in cell cycle progression (**Figure 6G**). These findings align with previous studies demonstrating that PPP1R8 is correlated with survival and cell proliferation in LGG (74). Notably, a similar effect has been observed in PPP1R8-deficient low-grade glioma cells, where reduced proliferation was linked to cell cycle arrest. Additionally, PPP1R8 deficiency resulted in an increase in cell apoptosis compared to the control group (**Figure 6H**). Taken together, our findings indicate that PPP1R8 plays a crucial role in promoting GBM cell proliferation, highlighting its potential as a therapeutic target in aggressive gliomas.

## 4 Discussion

GBM remains one of the most aggressive and challenging malignancies, with a highly complex TME that plays a central role in its progression and resistance to treatment (75, 76). A key feature of GBM is its ability to evade immune surveillance (77, 78), which contributes to its aggressive nature and poor prognosis. In this study, we identified IERGs as potential prognostic biomarkers in GBM and developed a predictive gene signature to assess patient survival. Leveraging CRISPR/Cas9 technology, we knocked out PPP1R8 in GBM cells and demonstrated that its loss significantly inhibited cell proliferation and promoted apoptosis. These findings highlight the pivotal role of IERGs in both immune evasion and GBM progression, suggesting their potential as prognostic indicators and therapeutic targets.

What sets this study apart is its integrative and multi-dimensional design. We combined large-scale public transcriptomic datasets, advanced statistical modeling, immune profiling, single-cell RNA sequencing analysis, and *in vitro* functional assays. Notably, we not only constructed and validated a robust immune escape-related gene signature across seven independent GBM cohorts, but also linked this signature to immunological characteristics, putative cellular origins, and experimentally confirmed phenotypes. This comprehensive approach spanning from population-level prognostic modeling to mechanistic cellular validation reinforces both the clinical relevance and biological plausibility of our findings, and underscores the translational potential of this work.

Through an in-depth analysis of the TCGA GBM dataset, we identified 36 IERGs linked to patient prognosis. Among them, 20 genes—including STAT2, IFNGR2, IFNAR1, and B2M—were associated with poorer survival, while 16 correlated with better outcomes. These findings suggest that the expression patterns of IERGs significantly influence GBM prognosis, as many of these genes are implicated in tumorigenesis. STAT2 is a key regulator of the STAT2/type I interferon axis, which governs tumor progression (67). Additionally, we identified a novel prognostic risk signature based on interferon response genes, which is closely associated with immune infiltration in glioblastoma (68, 69). Similarly, IFNGR2 has been linked to glioma prognosis and response to immune checkpoint blockade (65). The upregulation of canonical interferon-gamma signaling has been correlated with GBM progression, and its expression within the tumor microenvironment plays a crucial role in overall survival, particularly in pediatric diffuse midline glioma (DMG) patients (70, 71). Furthermore, glioma patients with lower B2M expression exhibited significantly longer survival than those with higher levels. Meta-analysis further established B2M as an independent prognostic marker in glioma, with moderate sensitivity in predicting the mesenchymal molecular subtype (79). In addition, B2M signaling in glioblastoma cells activates the PI3K/AKT/MYC/TGFβ1 axis, which sustains cancer stem cell properties and promotes M2-like macrophage polarization (80). These findings underscore the potential of IERGs as prognostic indicators and provide insights into their role in glioma pathophysiology.

We further developed a prognostic signature based on three key IERGs—STAT2, IFNGR2, and PPP1R8—using a multivariate Cox regression model. This signature, represented by the IEScore, was capable of stratifying GBM patients into high- and low-risk groups. Kaplan-Meier analysis confirmed that patients with higher IEScores had a substantially lower median OS compared to those in the low-risk group, validating the prognostic power of the signature. The predictive accuracy of the IEScore was further supported by AUC values. These results suggest that the IERGs-based signature offers strong predictive value and could complement existing clinical biomarkers, such as MGMT promoter methylation status, in guiding treatment decisions for GBM patients. Moreover, PCA demonstrated clear separation between the high- and low-risk groups, further confirming the reliability and robustness of the signature. In addition to its prognostic capabilities, the IEScore was shown to be independent of other clinicopathological features, such as MGMT status, IDH1 mutation, and age, which are known to influence GBM outcomes (81–83). Multivariate Cox regression analysis confirmed that the IEScore independently predicted survival outcomes, suggesting its potential as a standalone biomarker. However, although the prognostic value of the IEScore was validated in the TCGA cohort, it did not achieve statistical significance in multivariate analyses of the external validation cohorts (CGGA and LeeY). While the IEScore demonstrated prognostic relevance across multiple independent datasets, its multivariate significance was inconsistent, likely reflecting the aggressive nature of GBM, cohort-specific clinical features, and the limited number of long-term survivors typical of these datasets. The reduced multivariate significance in external cohorts may be largely explained by substantial clinical and molecular heterogeneity. As detailed in **Supplementary Table S1**, the CGGA and LeeY cohorts differ from the TCGA training set with respect to recurrence status, treatment exposure, IDH1 mutation frequency, G-CIMP status, and molecular subtype distribution. These variables critically influence tumor biology, the immune microenvironment, and patient outcomes, which can modulate the prognostic performance of immune-related signatures. Furthermore, incomplete clinical data, such as the absence of treatment information in the LeeY cohort, may hinder comprehensive adjustment for confounders in multivariate models. Collectively, these observations highlight the importance of accounting for cohort-specific characteristics when evaluating and interpreting prognostic models across independent GBM populations.

A key aspect of this study was the analysis of the TIME to understand how IERGs might influence immune cell infiltration in GBM. Our analysis revealed distinct immune cell profiles between the high- and low-risk groups. In the low-risk group, we observed higher levels of CD4^+^ effector memory T cells, CD8^+^ T cells, and natural killer (NK) cells, all of which are typically associated with anti-tumor immunity (84–86). In contrast, the high-risk group exhibited an increase in immunosuppressive cell types, such as macrophages, myeloid-derived suppressor cells (MDSCs), and mast cells, which have been linked to immune evasion and tumor progression in GBM (87–89). The similar trend of immunological differences observed between high- and low-risk groups across multiple cohorts and deconvolution platforms highlight the biological significance of IEScore as a marker of immune escape. Similarly to CIBERSORT and xCell, EPIC and TIMER analyses both demonstrated that high-risk tumors harbor higher levels of CD4^+^ T cells, cancer-associated fibroblasts, neutrophils, dendritic cells, and macrophages, indicative of a more immune-suppressive microenvironment and tumor-promoting contexture. The convergence of findings from CIBERSORT, xCell, EPIC, and TIMER strengthens the validity of our observations and underscores the relevance of IEScore in shaping the tumor immune microenvironment. These findings may have implications for predicting immunotherapy responses and developing combinatorial strategies for glioblastoma treatment (15, 90, 91).

Furthermore, the IEScore demonstrated utility beyond GBM by being applied to LGG, which are more commonly observed in younger patients. In the TCGA-LGG dataset and other independent LGG cohorts, the signature effectively stratified patients into high- and low-risk groups, with significant differences in OS observed between the two groups. The AUC values for survival prediction were consistently high across multiple LGG datasets, indicating the broader applicability of the IERGs signature in glioma prognosis. These findings are in line with prior research suggesting that immune escape mechanisms are not limited to GBM but also contribute to the progression of LGG (92, 93). This suggests that the IERGs signature could potentially serve as a valuable tool for the prognosis of a wide range of gliomas, helping clinicians make more informed decisions regarding treatment and patient management. In addition, our study revealed that while the prognostic signature effectively stratifies glioma patients by survival, it does not correlate with tumor grade, as evidenced by the similar distribution of LGG and GBM cases across risk groups and the lack of differential expression of IERGs between the two. This suggests that the signature reflects immune-related prognostic factors rather than histological malignancy. These findings highlight that immune evasion mechanisms may operate across glioma grades and contribute to poor outcomes independently of tumor aggressiveness.

PPP1R8 (also known as NIPP1) is a regulatory subunit of protein phosphatase 1 (PP1) and plays a multifaceted role in cellular processes, including gene expression, RNA processing, and cell cycle regulation (94). Previous studies have demonstrated that PPP1R8 modulates PP1 activity and influences chromatin structure and DNA repair mechanisms, particularly through its interaction with histone-modifying enzymes and splicing factors (95–97). Additionally, PPP1R8 has been implicated in tumorigenesis through its role in cell proliferation and survival in several cancers, such as Kidney Renal Clear Cell Carcinoma and breast cancer (98–100). However, its specific function in GBM remains largely unexplored. It exerts its regulatory effects by binding to PP1 and inhibiting its phosphatase activity within the nucleus. Emerging evidence suggests that PP1/PPP1R8 functions as a molecular regulator of directed cell migration by upregulating Cdc42 signaling, potentially enhancing the migratory properties of cancer cells (101). Additionally, the PP1 complex plays a crucial role in mitotic regulation, as its inhibition leads to mitotic arrest, activation of the spindle assembly checkpoint, and impaired checkpoint silencing, ultimately resulting in apoptosis or binucleation. This effect is likely mediated by PPP1R8, which sequesters PP1 from its mitotic interactors, potentially suppressing tumor growth (102). Furthermore, hypoxia-induced activation of PPP1R8 has been linked to enhanced metastatic potential and poor prognosis in hepatocellular carcinoma, suggesting a role for PPP1R8 in cancer progression (103). Additionally, PPP1R8 may contribute to carcinogenesis by enhancing DNA repair capacity and promoting resistance to genotoxic stress (104).

To further investigate the role of PPP1R8 in GBM, we used CRISPR/Cas9 to delete PPP1R8 in U251 GBM cells. Our findings revealed that PPP1R8 deficiency significantly reduced cell proliferation, as demonstrated by CCK-8 assay. Furthermore, cell cycle analysis indicated that PPP1R8 knockdown induced G2/S/M phase arrest, aligning with previous studies showing that protein phosphatase inhibitors can trigger apoptosis (105). Consistently, we observed that PPP1R8 deficiency increased apoptosis in GBM cells compared to wild-type controls. Together, these findings suggest that PPP1R8 promotes GBM cell proliferation and survival, potentially through its role in regulating DNA repair and intracellular signaling pathways. Further in-depth *in vitro* and *in vivo* studies are warranted to delineate the molecular mechanisms by which PPP1R8 contributes to glioblastoma progression.

## 5 Study limitations

Despite its promising findings, this study has several limitations. First, while the IERG-based prognostic signature was validated in independent datasets, the sample sizes were relatively not large, which may limit the generalizability of the results. Additionally, the validation cohorts did not fully replicate the multivariate analysis results observed in the TCGA dataset, likely due to the aggressive nature of GBM and the limited number of long-term survivors in these cohorts. Furthermore, while we identified significant differences in the TME between high- and low-risk groups, the mechanistic basis for these differences remains unclear, warranting further functional studies to elucidate the role of specific immune cell subsets. Although we analyzed the association of IEScore with chemotherapy and radiotherapy in the CGGA cohort and observed no significant differences, the influence of immunotherapy could not be assessed due to lack of relevant data. As treatment strategies for GBM evolve, future studies incorporating detailed therapeutic information, including immunotherapeutic interventions, are needed to refine the predictive utility of the IEScore. Finally, our functional experiments identified PPP1R8 as a potential tumorigenic driver in GBM, However, the underlying molecular mechanisms by which PPP1R8 and other signature genes contribute to immune evasion remain incompletely understood. Future studies such as rescue experiments, validation in a second patient-derived GBM model, and integrative transcriptomic and proteomic analyses to dissect interactions with immune cell populations and regulatory networks within the TME, will be essential to further strengthen and expand upon our findings.

## 6 Conclusion

This study demonstrates that IERGs are closely associated with GBM prognosis. We developed the IEScore, a prognostic signature based on the expression of STAT2, IFNGR2, and PPP1R8, which proved to be a reliable and robust predictor of patient outcomes across multiple datasets. The IEScore shows promise for clinical application in patient stratification and decision-making, including potential use in clinical trials. Our findings also offer new insights into the immune landscape of GBM, emphasizing the critical role of the TME in disease progression and identifying novel targets for immunotherapy. We observed that PPP1R8 deficiency impairs GBM cell proliferation and promotes apoptosis, warranting further mechanistic exploration. Future studies should aim to validate the IEScore in larger and more diverse patient populations and investigate its utility in guiding personalized immunotherapeutic approaches for GBM.

## Data Availability

The original contributions presented in the study are included in the article/**Supplementary Material**. Further inquiries can be directed to the corresponding author.

## Supplementary material

Supplementary Figure 1 Study workflow.

Supplementary Figure 2 Gene functional enrichment analysis of OS related immune escape related genes. **(A)** GO term analysis. **(B)** KEGG pathway analysis.

Supplementary Figure 3 Expression of signature genes in normal and tumor patients, as well as in low- and high-risk groups. **(A)** STAT2/IFNGR2/PPP1R8 expression in normal and GBM patients from TCGA database. STAT2/IFNGR2/PPP1R8 expression in low- and high-risk groups in **(B)** TCGA-GBM dataset, **(C)** CGGA-GBM dataset, **(D)** LeeY-GBM dataset.

Supplementary Figure 4 Predictive performance changes over time of IEScore and two published Interferon-related gene signatures using time-dependent AUC analysis.

Supplementary Figure 5 Association of the signature with clinical features from TCGA-GBM dataset. **(A)** Recurrence. **(B)** age. **(C)** Gender. **(D)** Subtype. **(E)** CIMP status. **(F)** IDH1 status. **(G)** MGMT status.

Supplementary Figure 6 Association of the signature with clinical features from CGGA-GBM dataset. **(A)** Recurrence. **(B)** age. **(C)** Gender. **(D)** Subtype. **(E)** IDH1 status. **(F)** CIMP status. **(G)** MGMT status. **(H)** Chemotherapy history. **(I)** Radiotherapy history.

Supplementary Figure 7 Association of the signature with clinical features from LeeY-GBM dataset. **(A)** Gender. **(B)** age. **(C)** MGMT status. **(D)** CIMP status.

Supplementary Figure 8 Association of the signature with tumor malignancy. **(A)** Kaplan-Meier curve of high- and low-risk patients in TCGA GBM and LGG combined cohorts. **(B)** Distribution of TCGA GBM and LGG patients across IERG-based risk groups.

Supplementary Figure 9 Tumor immune microenvironment analysis in CGGA-GBM dataset. **(A)** Infiltrated immune cell subsets in patients between low- and high-risk groups analyzed by xCell. **(B)**. Infiltrated myeloid cell subsets in patients between low- and high-risk groups analyzed by xCell. **(C)**. Infiltrated stromal cell subsets in patients between low- and high-risk groups analyzed by xCell.

Supplementary Figure 10 Tumor immune microenvironment analysis in LeeY-GBM dataset. **(A)**. Infiltrated immune cell subsets in patients between low- and high-risk groups analyzed by xCell. **(B)**. Infiltrated myeloid cell subsets in patients between low- and high-risk groups analyzed by xCell. **(C)**. Infiltrated stromal cell subsets in patients between low- and high-risk groups analyzed by xCell.

Supplementary Figure 11 Tumor immune microenvironment analysis by CIBERSORT. **(A)**. Infiltrated immune cell subsets in patients between low- and high-risk groups in TCGA-GBM dataset. **(B)**. Infiltrated immune cell subsets in patients between low- and high-risk groups in CGGA-GBM dataset. **(C)**. Infiltrated immune cell subsets in patients between low- and high-risk groups in LeeY-GBM dataset.

Supplementary Figure 12 Immune cell composition differences between high- and low-risk groups across independent deconvolution algorithms by TIMER and EPIC. **(A)** Immune cell fractions estimated by TIMER in TCGA_GBM, CGGA_GBM, and LeeY_GBM cohorts. **(B)** Immune cell fractions estimated by EPIC in the same cohorts. Violin plots show the distribution of immune cell types between high- and low-risk groups. Statistical differences were assessed using Wilcoxon rank-sum test. c(0, 0.001, 0.01, 0.05, 1)p-values are shown as “***” p<0.001, “**” p<0.01, “*” p<0.05, “ “ p>0.05).

Supplementary Figure 13 Expression of signature genes in NCI-60 tumor cell lines and validation of PPP1R8 knockout. **(A)** Expression profiles of the signature genes across NCI-60 tumor cell lines. **(B)** Western blot analysis confirming PPP1R8 knockout in U251 cells using CRISPR/Cas9-mediated gene editing.

Supplementary Table 1 GBM datasets for Development and validation of a prognostic immune escape related gene signature.

Supplementary Table 2 Immune escape related gene list.

Supplementary Table 3 Significantly enriched KEGG pathways associated with overall survival-related immune escape genes.

Supplementary Table 4 Comparison of predictive performance between IEScore and two published Interferon-related gene signatures using time-dependent AUC analysis.
