# Supplementary figures and images for: Immune intrinsic escape signature stratifies prognosis, characterizes the tumor immune microenvironment, and identifies tumorigenic PPP1R8 in glioblastoma multiforme patients
Source: Front Immunol. 2025 Aug 6;16:1577920. doi: 10.3389/fimmu.2025.1577920 (PMC12364687; doi:10.3389/fimmu.2025.1577920)

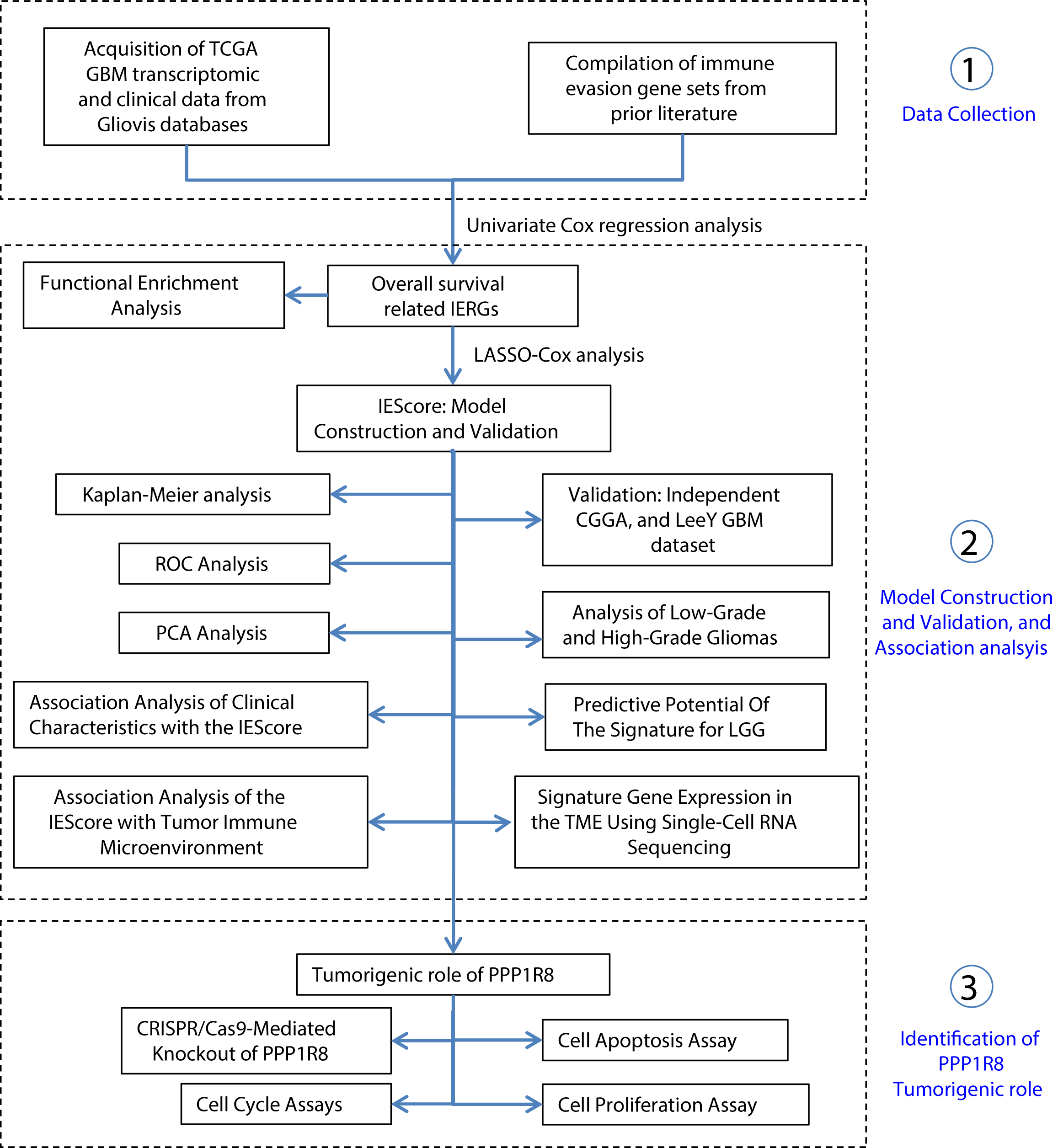

Supplement: Supplementary Figure 1 — Study workflow. [file Image1.tif]

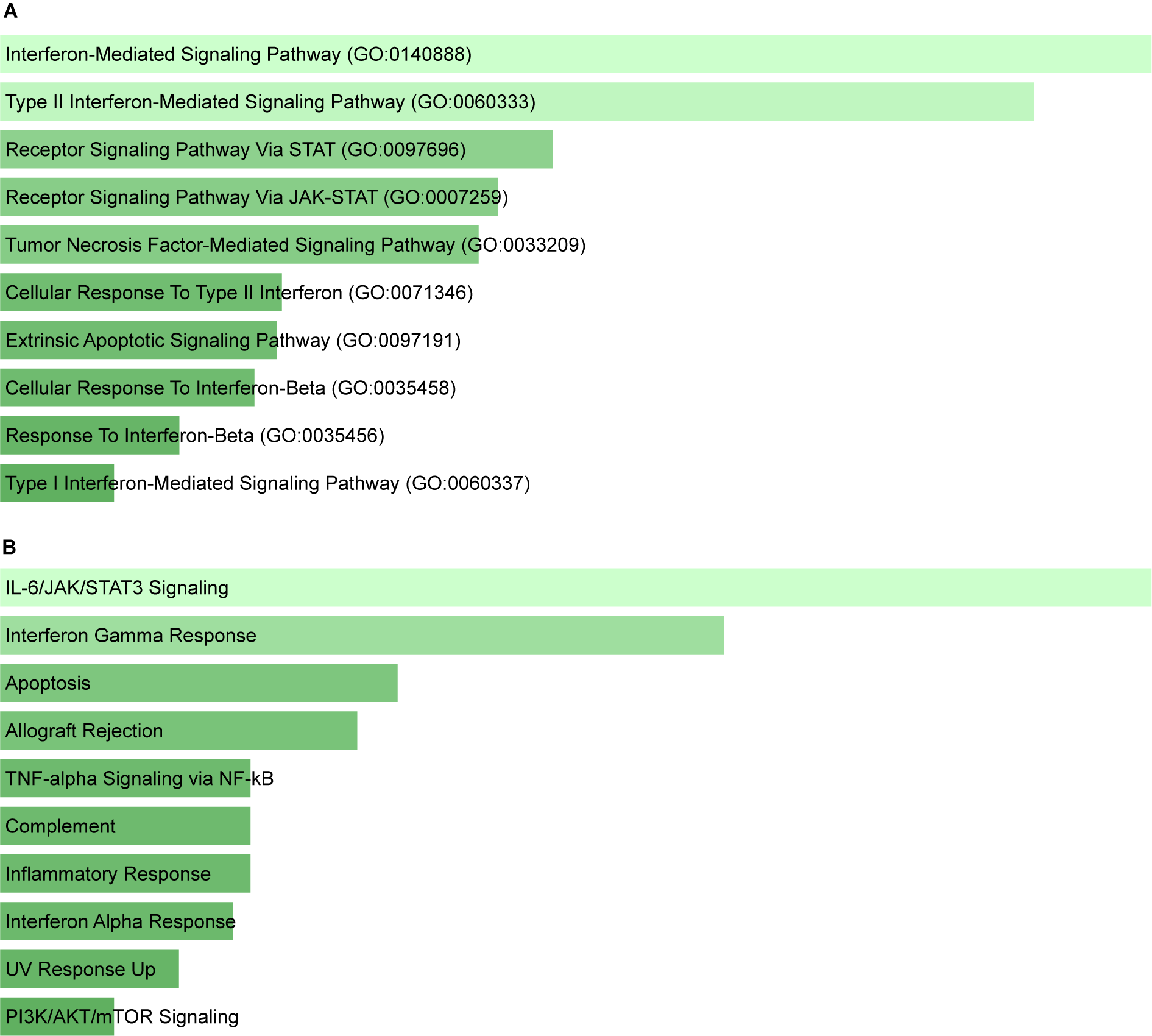

Supplement: Supplementary Figure 2 — Gene functional enrichment analysis of OS related immune escape related genes. (A) GO term analysis. (B) KEGG pathway analysis. [file Image2.tif]

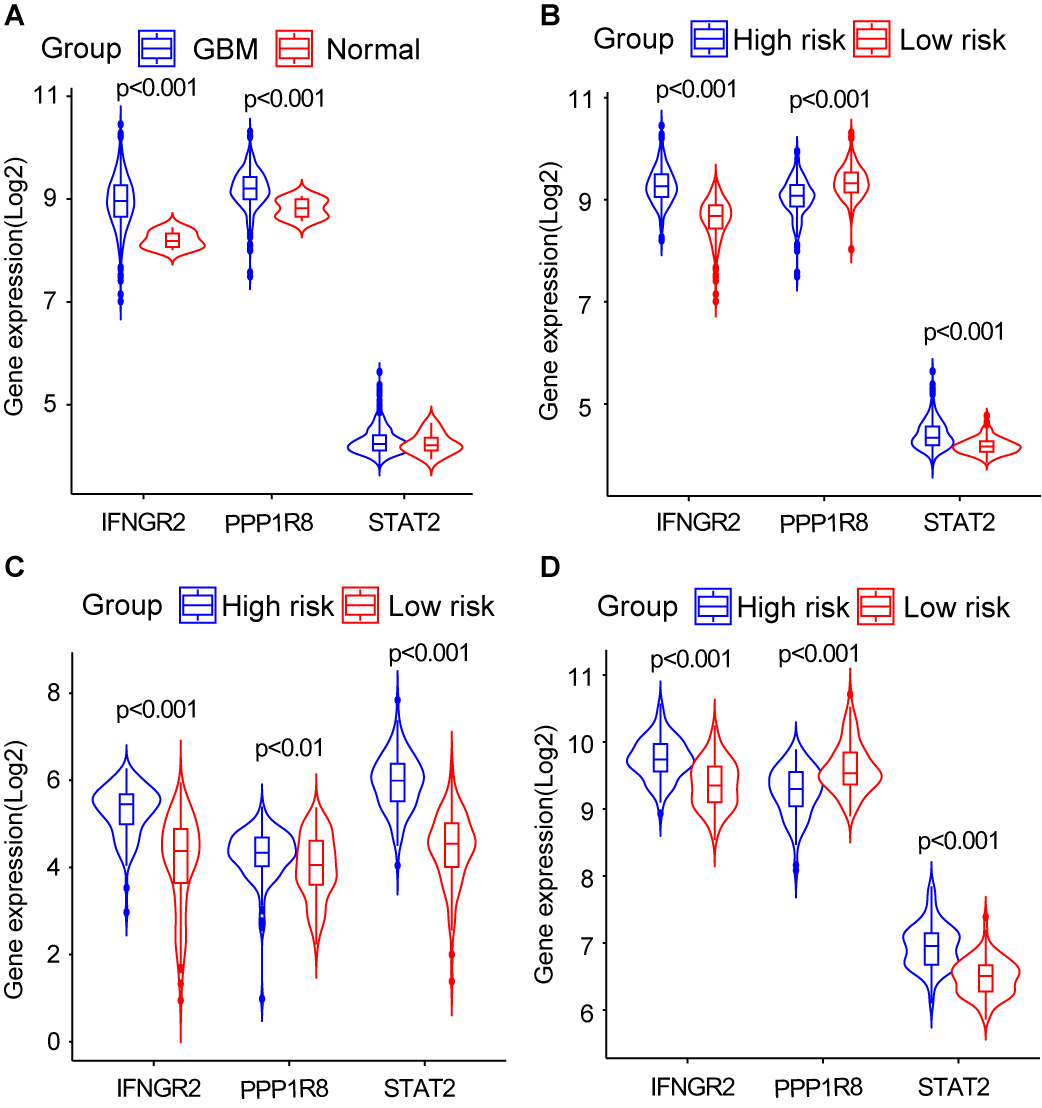

Supplement: Supplementary Figure 3 — Expression of signature genes in normal and tumor patients, as well as in low- and high-risk groups. (A) STAT2/IFNGR2/PPP1R8 expression in normal and GBM patients from TCGA database. STAT2/IFNGR2/PPP1R8 expression in low- and high-risk groups in (B) TCGA-GBM dataset, (C) CGGA-GBM dataset, (D) LeeY-GBM dataset. [file Image3.tif]

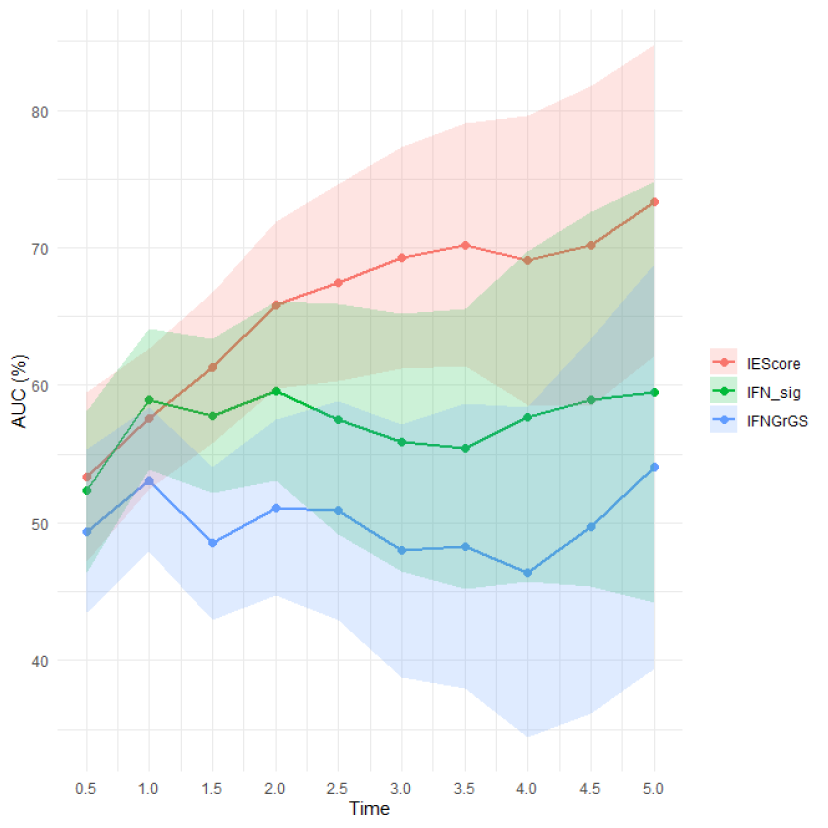

Supplement: Supplementary Figure 4 — Predictive performance changes over time of IEScore and two published Interferon-related gene signatures using time-dependent AUC analysis. [file Image4.tif]

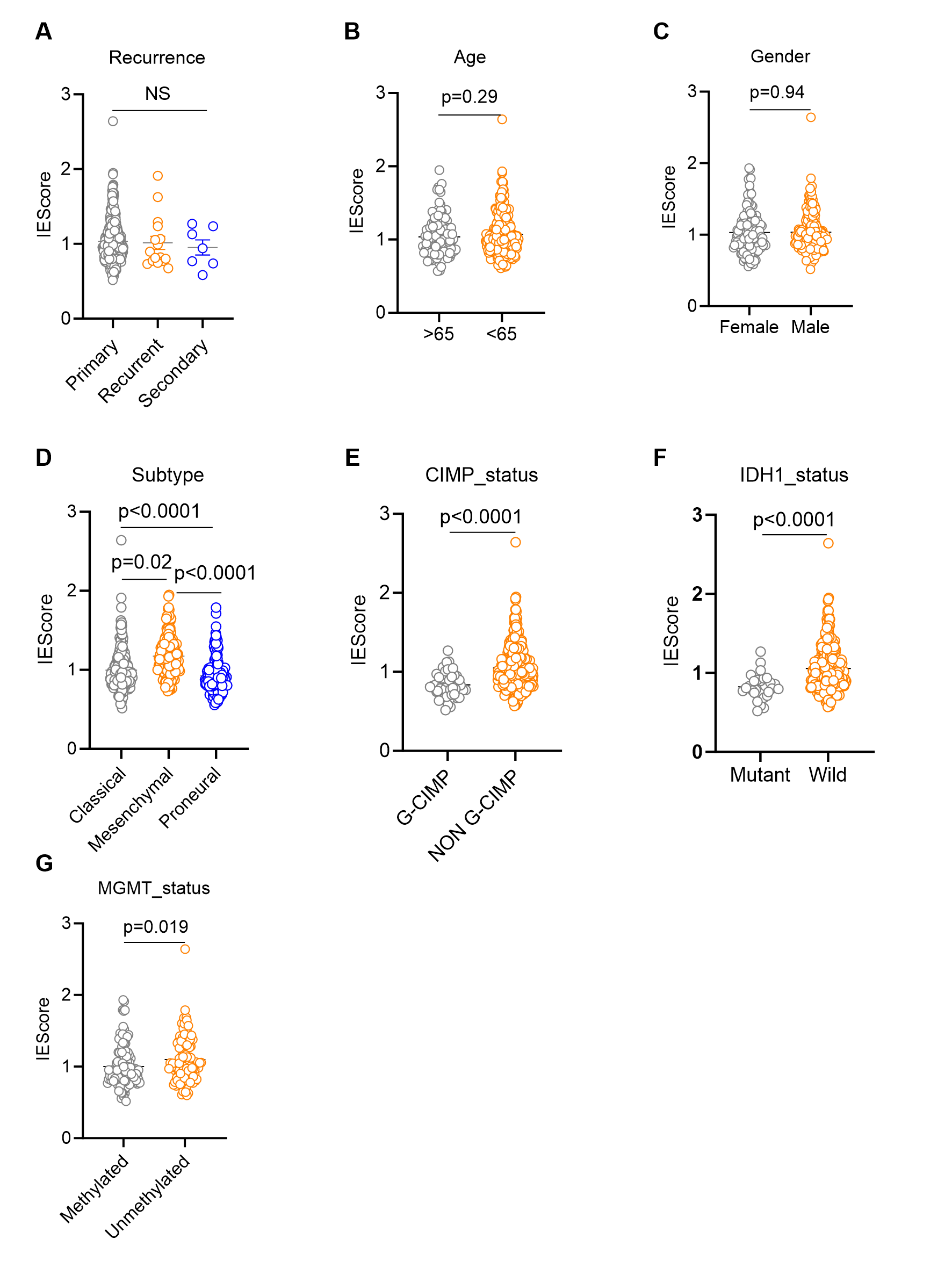

Supplement: Supplementary Figure 5 — Association of the signature with clinical features from TCGA-GBM dataset. (A) Recurrence. (B) age. (C) Gender. (D) Subtype. (E) CIMP status. (F) IDH1 status. (G) MGMT status. [file Image5.tif]

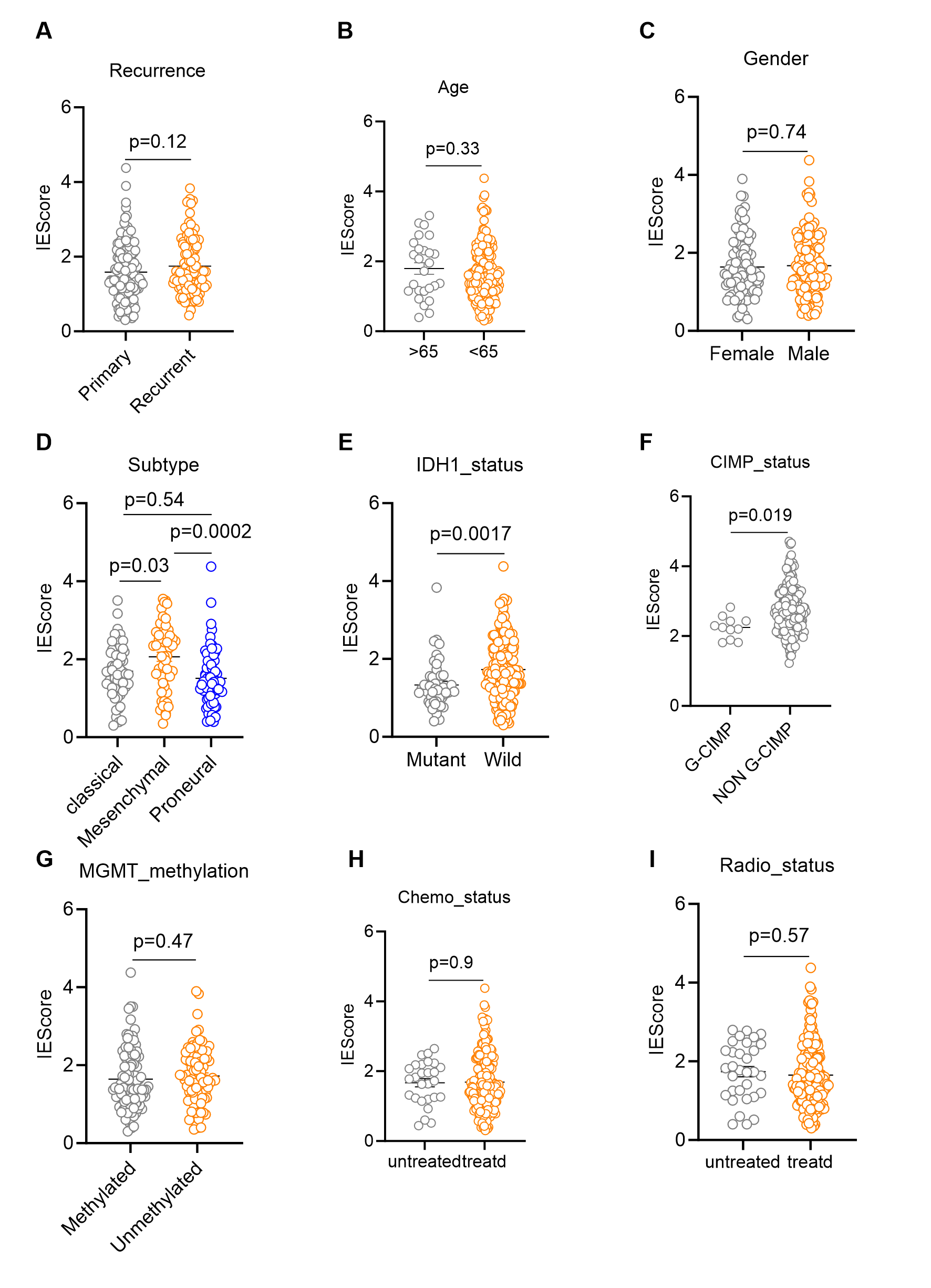

Supplement: Supplementary Figure 6 — Association of the signature with clinical features from CGGA-GBM dataset. (A) Recurrence. (B) age. (C) Gender. (D) Subtype. (E) IDH1 status. (F) CIMP status. (G) MGMT status. (H) Chemotherapy history. (I) Radiotherapy history. [file Image6.tif]

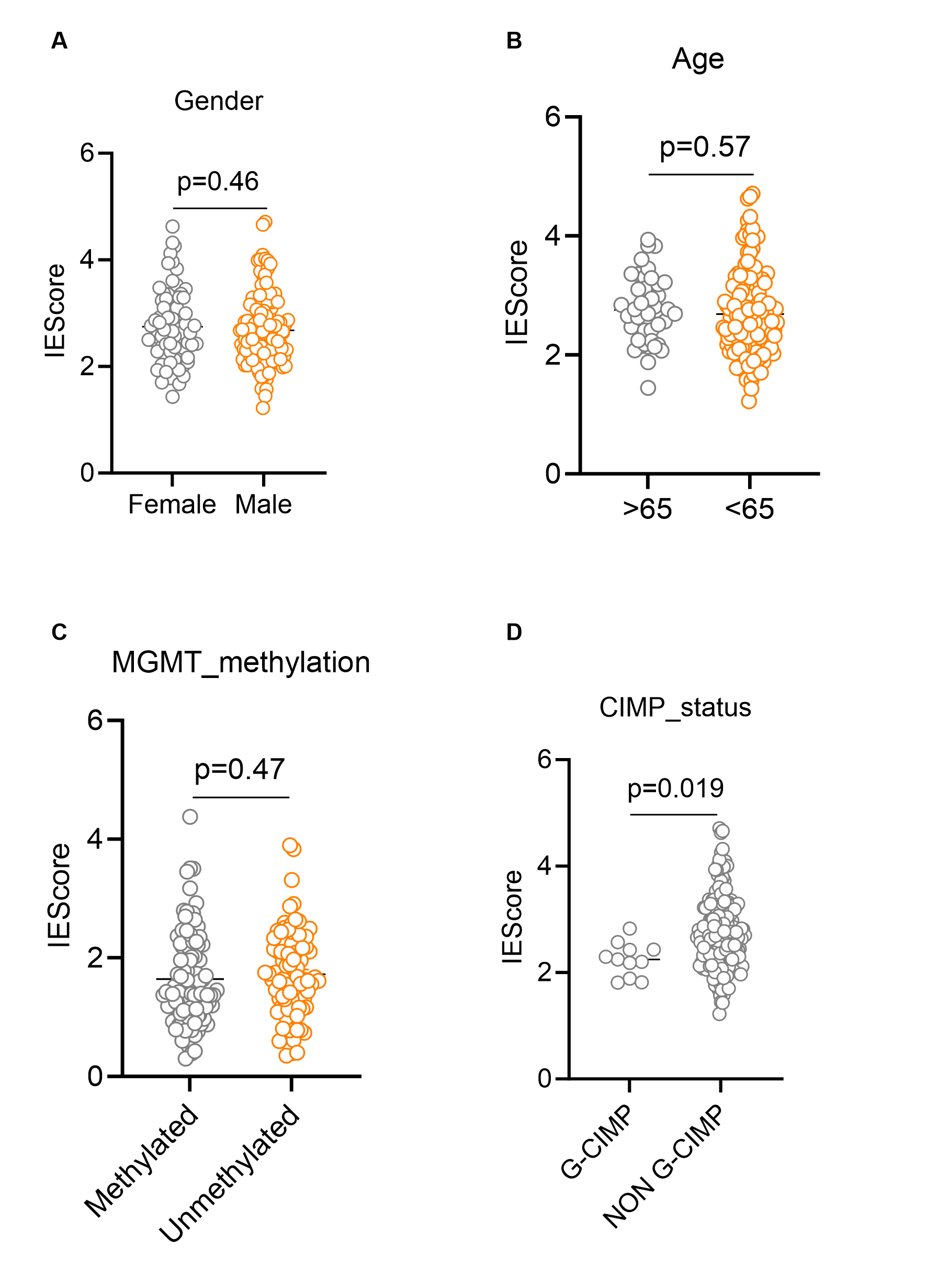

Supplement: Supplementary Figure 7 — Association of the signature with clinical features from LeeY-GBM dataset. (A) Gender. (B) age. (C) MGMT status. (D) CIMP status. [file Image7.tif]

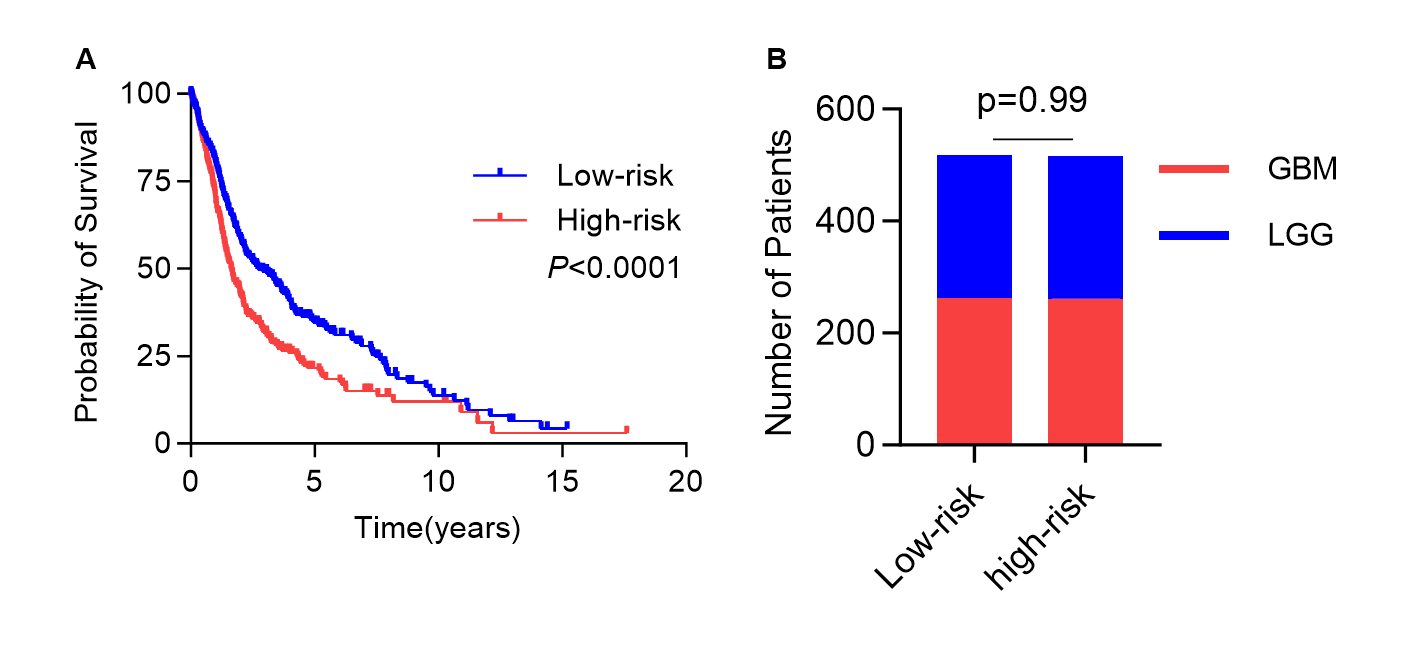

Supplement: Supplementary Figure 8 — Association of the signature with tumor malignancy. (A) Kaplan-Meier curve of high- and low-risk patients in TCGA GBM and LGG combined cohorts. (B) Distribution of TCGA GBM and LGG patients across IERG-based risk groups. [file Image8.tif]

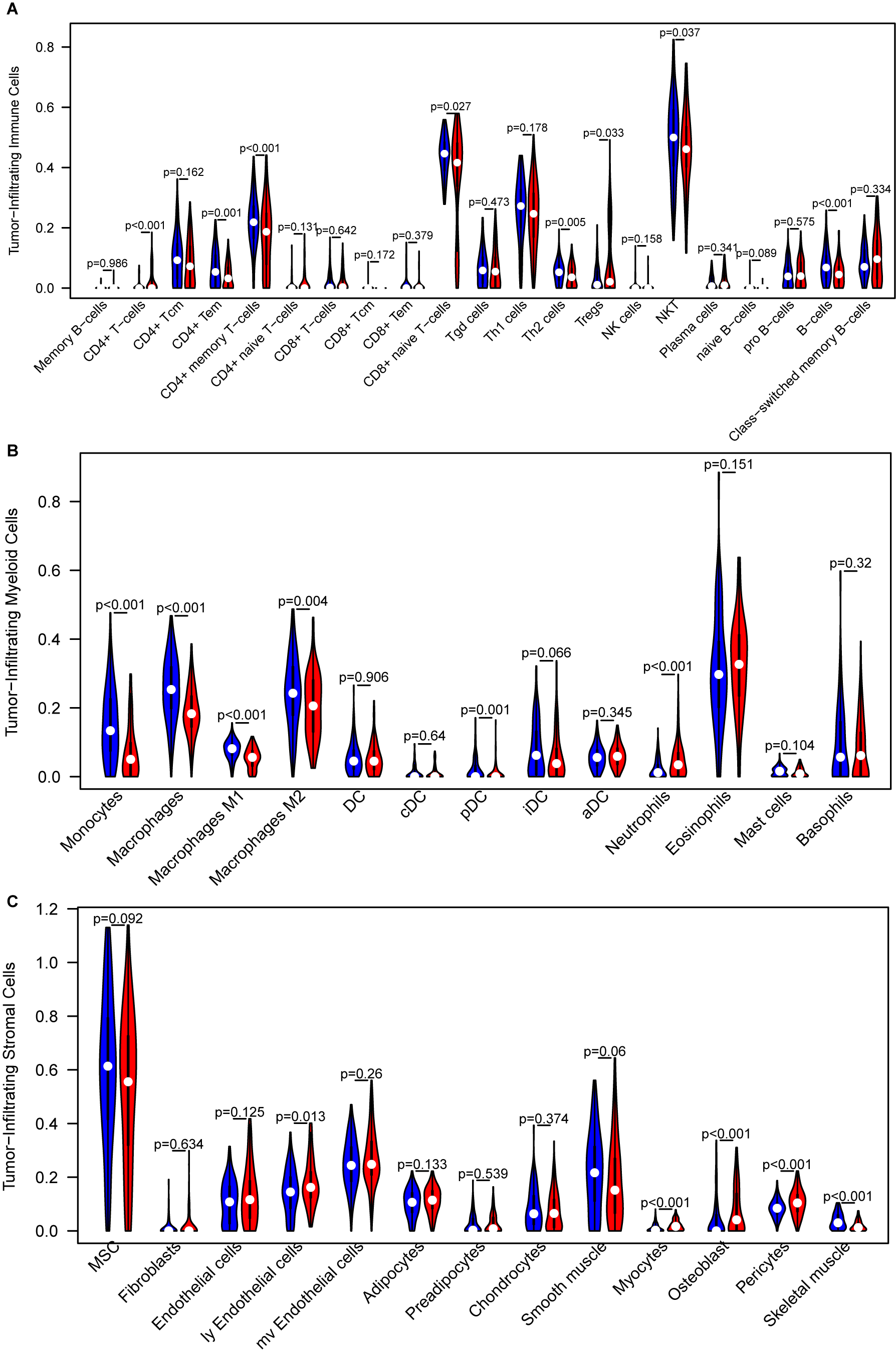

Supplement: Supplementary Figure 9 — Tumor immune microenvironment analysis in CGGA-GBM dataset. (A) Infiltrated immune cell subsets in patients between low- and high-risk groups analyzed by xCell. (B). Infiltrated myeloid cell subsets in patients between low- and high-risk groups analyzed by xCell. (C). Infiltrated stromal cell subsets in patients between low- and high-risk groups analyzed by xCell. [file Image9.tif]

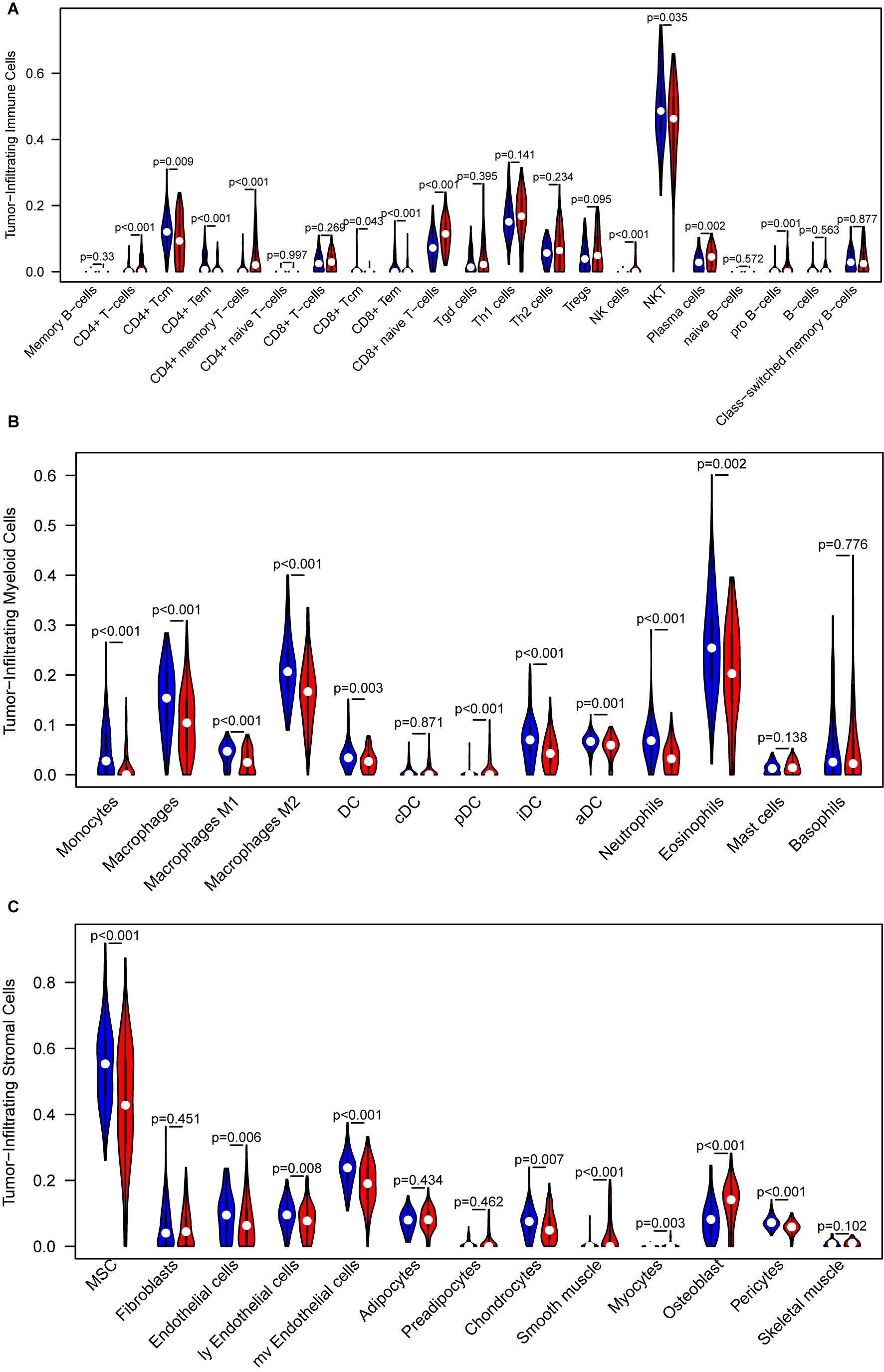

Supplement: Supplementary Figure 10 — Tumor immune microenvironment analysis in LeeY-GBM dataset. (A). Infiltrated immune cell subsets in patients between low- and high-risk groups analyzed by xCell. (B). Infiltrated myeloid cell subsets in patients between low- and high-risk groups analyzed by xCell. (C). Infiltrated stromal cell subsets in patients between low- and high-risk groups analyzed by xCell. [file Image10.tif]

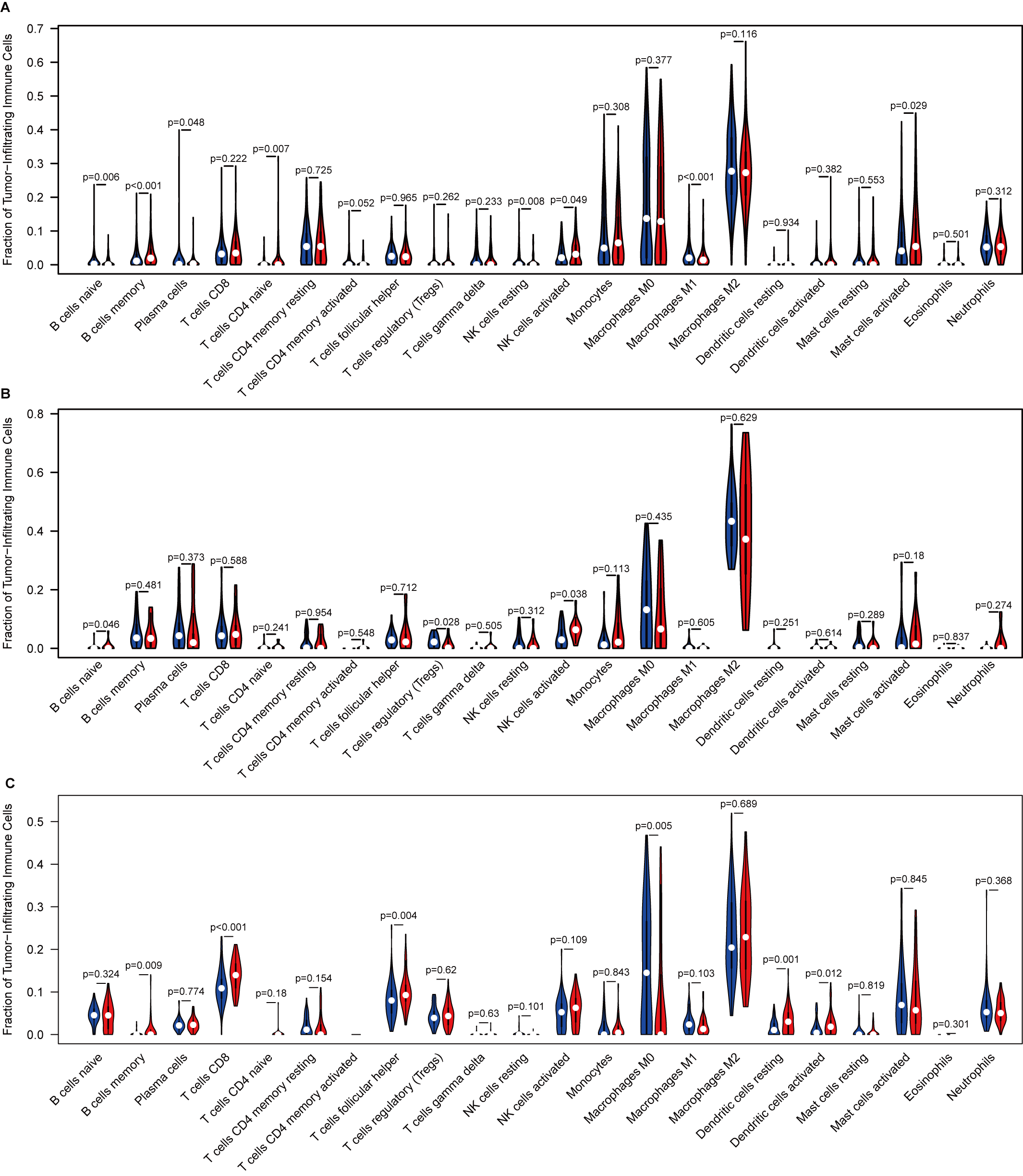

Supplement: Supplementary Figure 11 — Tumor immune microenvironment analysis by CIBERSORT. (A). Infiltrated immune cell subsets in patients between low- and high-risk groups in TCGA-GBM dataset. (B). Infiltrated immune cell subsets in patients between low- and high-risk groups in CGGA-GBM dataset. (C). Infiltrated immune cell subsets in patients between low- and high-risk groups in LeeY-GBM dataset. [file Image11.tif]

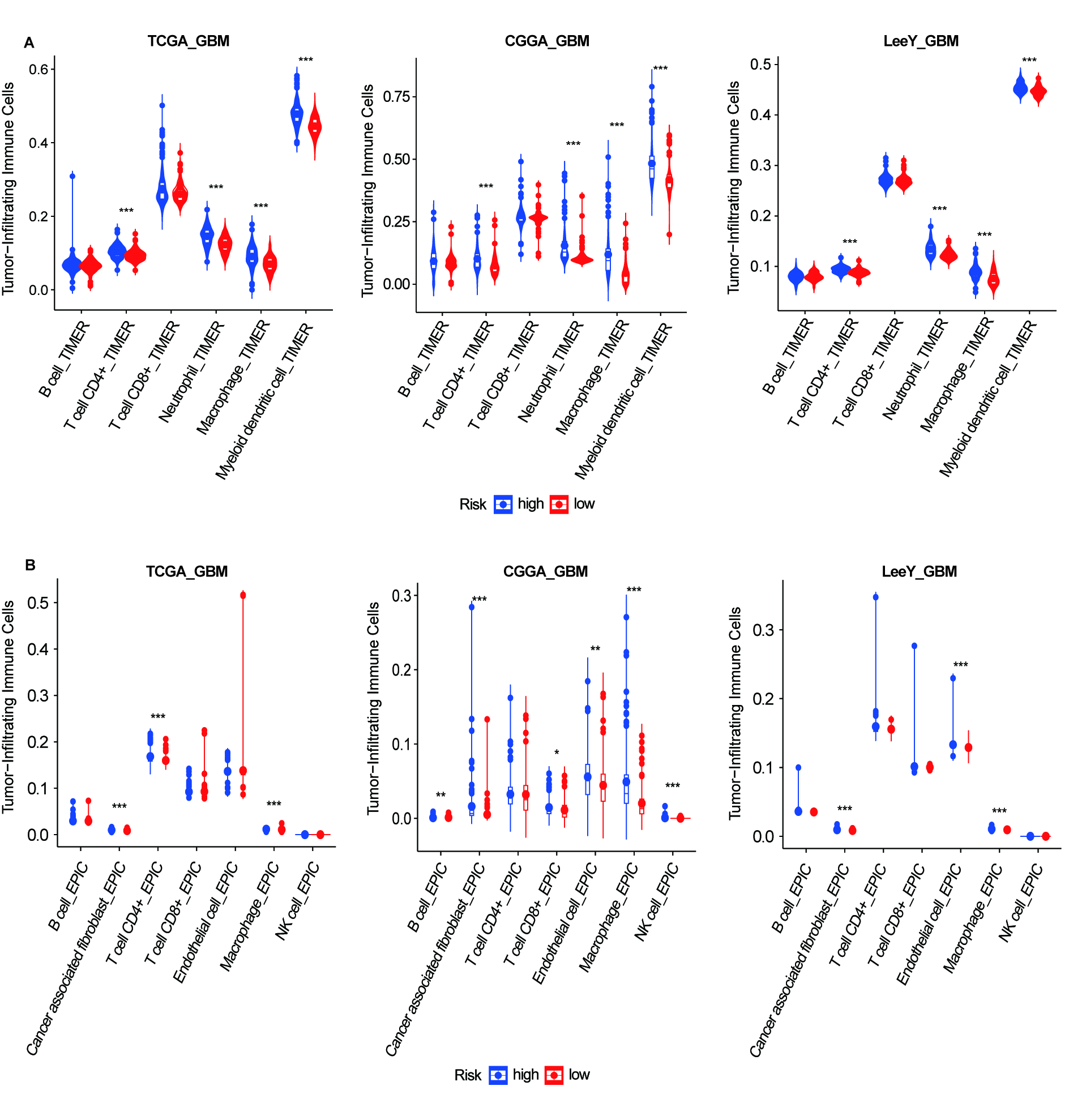

Supplement: Supplementary Figure 12 — Immune cell composition differences between high- and low-risk groups across independent deconvolution algorithms by TIMER and EPIC. (A) Immune cell fractions estimated by TIMER in TCGA_GBM, CGGA_GBM, and LeeY_GBM cohorts. (B) Immune cell fractions estimated by EPIC in the same cohorts. Violin plots show the distribution of immune cell types between high- and low-risk groups. Statistical differences were assessed using Wilcoxon rank-sum test. c(0, 0.001, 0.01, 0.05, 1)p-values are shown as “***” p<0.001, “**” p<0.01, “*” p<0.05, “ “ p>0.05). [file Image12.tif]

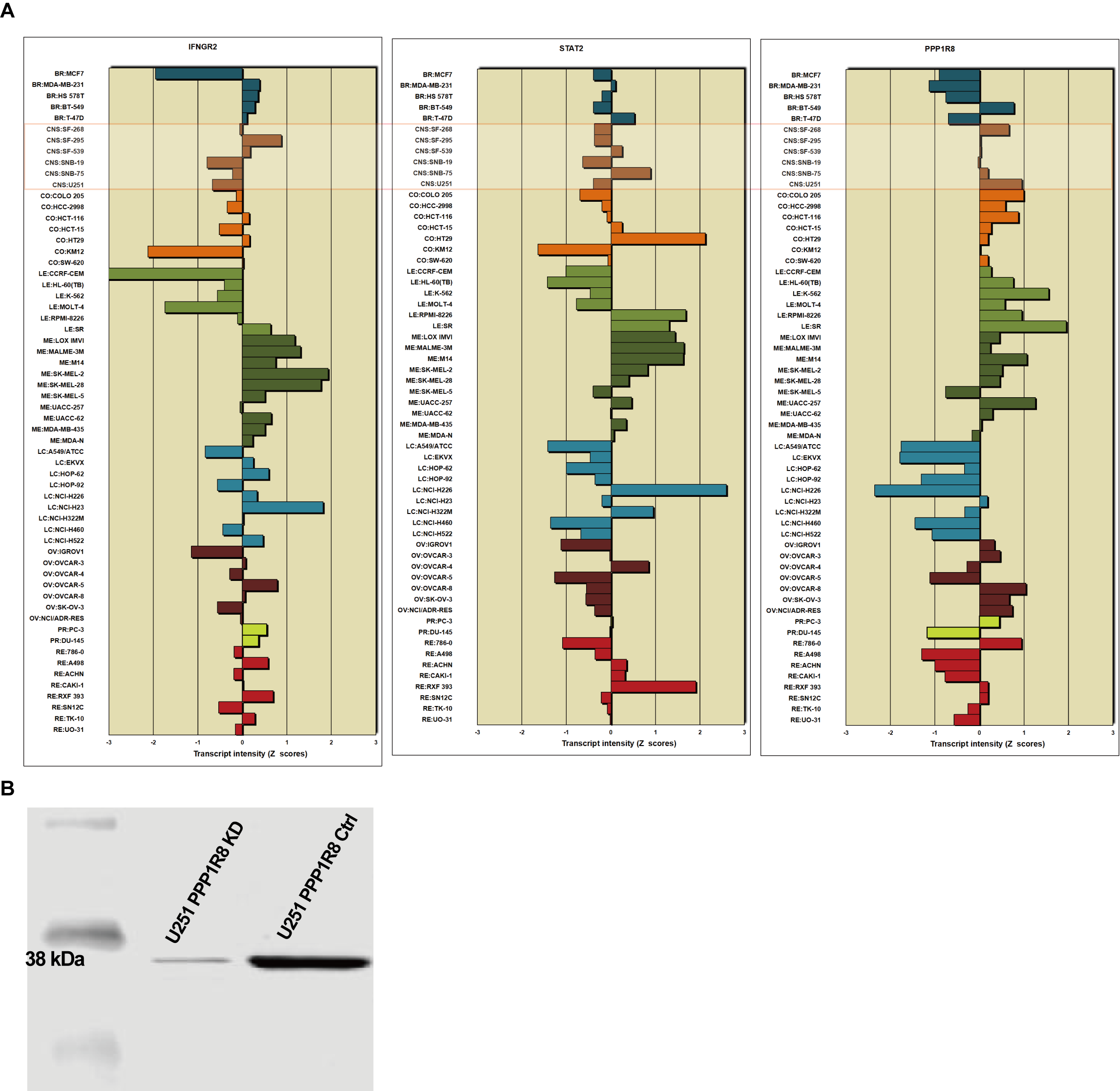

Supplement: Supplementary Figure 13 — Expression of signature genes in NCI-60 tumor cell lines and validation of PPP1R8 knockout. (A) Expression profiles of the signature genes across NCI-60 tumor cell lines. (B) Western blot analysis confirming PPP1R8 knockout in U251 cells using CRISPR/Cas9-mediated gene editing. [file Image13.tif]
