# Supplementary material for: Immune intrinsic escape signature stratifies prognosis, characterizes the tumor immune microenvironment, and identifies tumorigenic PPP1R8 in glioblastoma multiforme patients
Source: Front Immunol. 2025 Aug 6;16:1577920. doi: 10.3389/fimmu.2025.1577920 (PMC12364687; doi:10.3389/fimmu.2025.1577920)
Supplement: Supplementary Table 1 — GBM datasets for Development and validation of a prognostic immune escape related gene signature. [file Table1.docx]

**Table S1. Clinical characteristics of GBM populations used in this study.**

| Type | Dataset | Clinical Feature | | Cases(n) |
| --- | --- | --- | --- | --- |
| Training set | TCGA_GBM  (Microarray) | Recurrence |  |  |
|  |  |  | Primary | 497 |
|  |  |  | Recurrent | 16 |
|  |  |  | Secondary | 7 |
|  |  |  | NA | 8 |
|  |  | Subtype |  |  |
|  |  |  | Classical | 198 |
|  |  |  | Mesechynal | 165 |
|  |  |  | Proneural | 162 |
|  |  |  |  |  |
|  |  | CIMP_Status |  |  |
|  |  |  | G-CIMP | 46 |
|  |  |  | Non G-CIMP | 479 |
|  |  | IDH1_Status |  |  |
|  |  |  | Wild | 372 |
|  |  |  | Mutant | 30 |
|  |  |  | NA | 126 |
|  |  | MGMT_Status |  |  |
|  |  |  | Unmethylated | 177 |
|  |  |  | Methylated | 170 |
|  |  |  | NA | 181 |
|  |  | Age |  |  |
|  |  |  | >65 | 175 |
|  |  |  | ≤65 | 344 |
|  |  |  | NA | 9 |
|  |  | Gender |  |  |
|  |  |  | Male | 314 |
|  |  |  | Female | 203 |
|  |  |  | NA | 11 |
|  |  | Vital |  |  |
|  |  |  | Live | 78 |
|  |  |  | Dead | 447 |
|  |  |  | NA | 3 |
| Validation set-1 | CGGA_GBM  (Bulk RNA-seq) | Recurrence |  |  |
|  |  |  | Primary | 133 |
|  |  |  | Recurrent | 104 |
|  |  | Subtype |  |  |
|  |  |  | Classical | 56 |
|  |  |  | Mesechynal | 53 |
|  |  |  | Proneural | 73 |
|  |  |  | NA | 55 |
|  |  | IDH1_Status |  |  |
|  |  |  | Wild | 182 |
|  |  |  | Mutant | 45 |
|  |  |  | NA | 10 |
|  |  | MGMT_Status |  |  |
|  |  |  | Unmethylated | 89 |
|  |  |  | Methylated | 104 |
|  |  | Age |  |  |
|  |  |  | >65 | 26 |
|  |  |  | ≤65 | 211 |
|  |  | Gender |  |  |
|  |  |  | Male | 139 |
|  |  |  | Female | 98 |
|  |  | Radio_status |  |  |
|  |  |  | Non treated | 24 |
|  |  |  | Treated | 192 |
|  |  |  | NA | 21 |
|  |  | Chemo_status |  |  |
|  |  |  | Non treated | 25 |
|  |  |  | Treated | 190 |
|  |  |  | NA | 22 |
|  |  | Vital |  |  |
|  |  |  | Live | 46 |
|  |  |  | Dead | 191 |
| Validation set-2 | LeeY_GBM  (Microarray) | Subtype |  |  |
|  |  |  | Classical | 66 |
|  |  |  | Mesechynal | 56 |
|  |  |  | Proneural | 69 |
|  |  | Age |  |  |
|  |  |  | >65 | 41 |
|  |  |  | ≤65 | 150 |
|  |  | Gender |  |  |
|  |  |  | Male | 117 |
|  |  |  | Female | 74 |
|  |  | CIMP_status |  |  |
|  |  |  | G-CIMP | 11 |
|  |  |  | Non G-CIMP | 180 |
|  |  | Vital |  |  |
|  |  |  | Live | 15 |
|  |  |  | Dead | 176 |
